# Co-payments and equity in care: enhancing hospitalisation policy for Palestine refugees in Lebanon

**DOI:** 10.1186/s12913-021-07427-8

**Published:** 2022-01-29

**Authors:** Sara Valente de Almeida, Gloria Paolucci, Akihiro Seita, Hala Ghattas

**Affiliations:** 1 Department of Infectious Disease Epidemiology, Imperial College London, London, UK; 2 Department of Health, United Nations Relief and Works Agency for Palestine Refugees in the Near East (UNRWA), Amman, Jordan; 3 Centre for Research in Population and Health, Faculty of Health Sciences, American University of Beirut, Beirut, Lebanon

**Keywords:** Refugees, Co-payments in healthcare, Global health

## Abstract

**Background:**

This paper measures the impact of introducing a 10% co-payment on secondary care hospitalization costs for Palestine refugees living in Lebanon (PRL) in all UNRWA contracted hospitals, except for the Red Crescent Society. This ex-post analysis provides a detailed insight on the direction and magnitude of the policy impact in terms of demand by hospital type, average length of stay and treatment costs.

**Methods:**

With a complete population episode level dataset, we use multinomial logit, negative binomial, and linear models to estimate impacts on the different dependent variables, controlling for disease, patient and hospital characteristics.

**Results:**

After the implementation patients were at least 4 pp (p <0.01) more likely to choose a Red Crescent Society hospital for secondary care, instead of one with co-payment. Average length of stay was not affected in general, despite the increase in control at all UNRWA contracted hospitals. Except for the decrease in UNRWA contribution, did not find a statistically significant impact of the co-payment on costs, nor for the provider or for the patient. Findings suggest that introducing a 10% co-payment for secondary hospital care had an impact on patients’ health care budget, leading to demand shifts towards cheaper options - i.e., patients had to chose care based on financial constraints rather than on their treatment preferences.

**Conclusion:**

Before changing healthcare payment schemes in different types of hospitals, facilities offering free of charge treatment should be assessed and prepared for potential demand shifts to avoid overcapacity and the collapse of health care services for such a fragile population. In addition, exemptions from co-payments should be considered for patients with severe health conditions and financial constraints, who, according to our results, are the most likely to change their pattern of care due to an increase in treatment costs.

**Supplementary Information:**

The online version contains supplementary material available at (10.1186/s12913-021-07427-8).

## Background

Palestine refugees are the oldest and one of the largest refugee groups in the world, having been displaced since 1949 and accounting for around 5.5 million people spread across Jordan, Lebanon, West Bank and Gaza [1]. Particularly in Lebanon, Palestine refugees are not recognized as citizens, living with extremely restricted access to the job market (not entitled to work in as many as 39 professions) and without property rights. The United Nations Relief and Works Agency for Palestine Refugees in the Near East (UNRWA) provides essential development and humanitarian assistance to Palestine refugees including education, primary health care, relief and social services, amongst other services. However UNRWA has faced financial challenges in the last few years [2–4].

The Lebanese healthcare system has been under increasing pressure since the Syria conflict, which started in 2011 and forced local communities to be displaced to the neighbouring countries, including Lebanon [5]. Implementing the most appropriate and sustainable payment schemes in healthcare is thus as complex as it is key to ensure general access to health care and healthy lives in this context.

In terms of secondary health care, UNRWA has historically covered health expenses of Palestine refugees through the partial reimbursement of costs, incurred at any contracted hospital (private, public, UNRWA and NGO hospitals). The amounts covered vary across operation areas and are managed at the local level by the Health Department of the respective field office or headquarters. In the beginning of 2016, due to severe budget constraints, UNRWA in Lebanon explored alternative health financing arrangements and implemented new policies adjusting the co-payment coverage scheme, reducing secondary care cost coverage from 100% to 90% in private and public hospitals, while maintaining all costs covered at the Palestine Red Crescent Society hospitals (PRCS).

This study goes into the details of this policy change and aims to shed light on its impact on demand and supply of healthcare. This work contributes to the literature on the effect of co-payments in healthcare with a complete population database in a limited resource context, and provides specific insights to inform policies to improve access to healthcare for Palestine refugees in Lebanon (PRL).

### UNRWA hospitalization policy changes: a natural experiment

The policy change of interest in this study had a long and complex path towards implementation. In January 2016, UNRWA increased tertiary care coverage from 50 to 60% and reduced secondary care coverage from 100% to 80% in private, 85% in public and 95% in PRCS hospitals. Additionally, by the end of February 2016, UNRWA announced the creation of a Medical Hardship Fund (MHF), a program designed to ensure access to treatment for those living in extreme poverty and suffering from catastrophic health conditions - including support at the secondary healthcare level (in 2016 the percentage of UNRWA hospitalization accessed by MHF was of 18.4*%* [6]). Nonetheless, under these new conditions most patients had to cover a larger share of their hospitalization costs out of pocket which raised strong concerns and led to protests against the Agency’s decision.

**Fig. 1:**
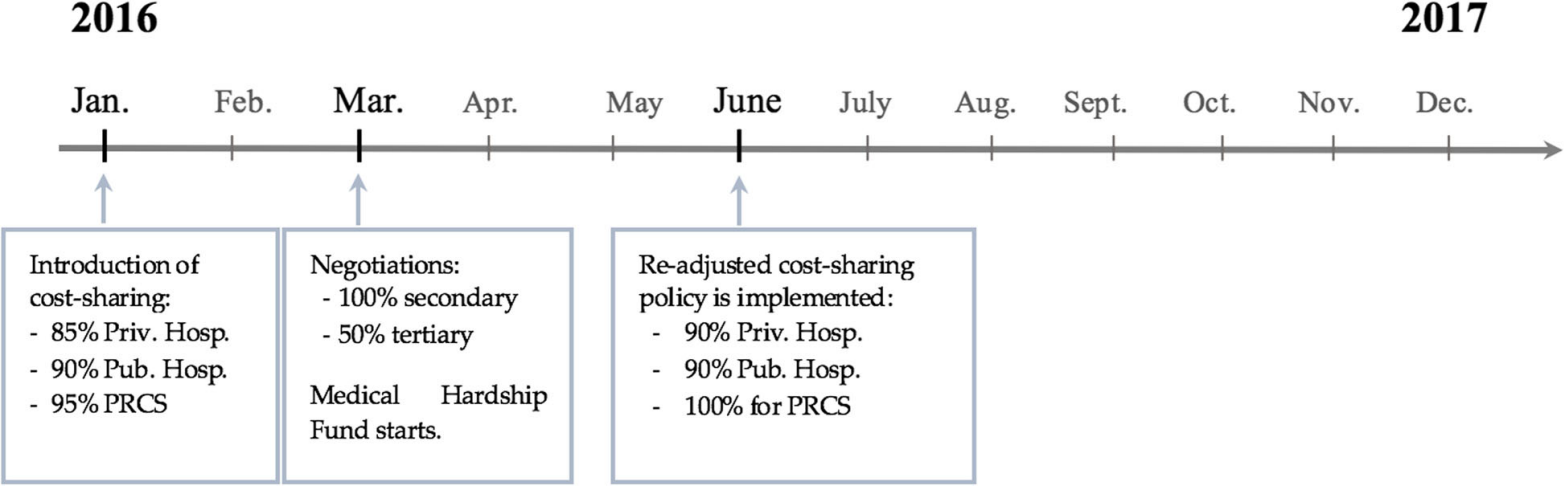
Policy timeline

UNRWA contracts services from thirty-five private hospitals, five Palestine Red Crescent Society and four public hospitals in Lebanon. Since the access to the most available hospitals became more expensive, users had less options for treatment - in 2016 the average cost of an appendectomy (surgical removal of the appendix) was around 734 USD in public and 683 USD in private hospitals. With UNRWA covering 90% this means the patient would still have to pay around 70 USD, which can be a significant cost for a family already in financial distress. The resulting tensions led UNRWA to open the matter to negotiations and suspend the cost-sharing policy between April and June 2016, changing coverage back to 100% for secondary care in all hospitals (as it had been until December 2015) [7]. This period gives us pre-policy implementation data to use as a natural experiment for the analysis. After the negotiations were concluded, UNRWA re-adjusted the policy to meet partially demands of the population. On June 1^*st*^ 2016, the percentage of the Agency’s coverage for secondary care was set to 90% for government and private hospitals and 100% for PRCS hospitals, maintaining the 60% coverage for tertiary care in all contracted hospitals (up to a ceiling of 5,200 USD per admission) (see Fig. 1). Together with this last policy, UNRWA revised the monitoring process for length of stay at the geographical area level (geographical areas of operation are formally defined by UNRWA). Each patient diagnosis and expected length of stay were confirmed by an Area Hospitalization Medical Officer (AHMO) (who produced an approval in accordance) and later extensions had to be approved by UNRWA. Unjustified stays were not covered by the institution, which provided an incentive for hospitals to comply.

The period after June 2016 will be equivalent to an experiment second-stage when we measure how the 10% co-payment changes demand between the ex post and ex ante stages.

UNRWA is the main official provider of health care for Palestine refugees and almost all refugees are accessing hospitals through UNRWA hospitalization support program. According to the last AUB Survey the overall health conditions of this population are fragile [8]. Namely, around 37% of the Palestine refugees from Lebanon (PRL) reported to be chronically ill and more likely to be hospitalized, with acute illness and disability percentages around 63% and 10.3*%*, respectively.

**Fig. 2:**
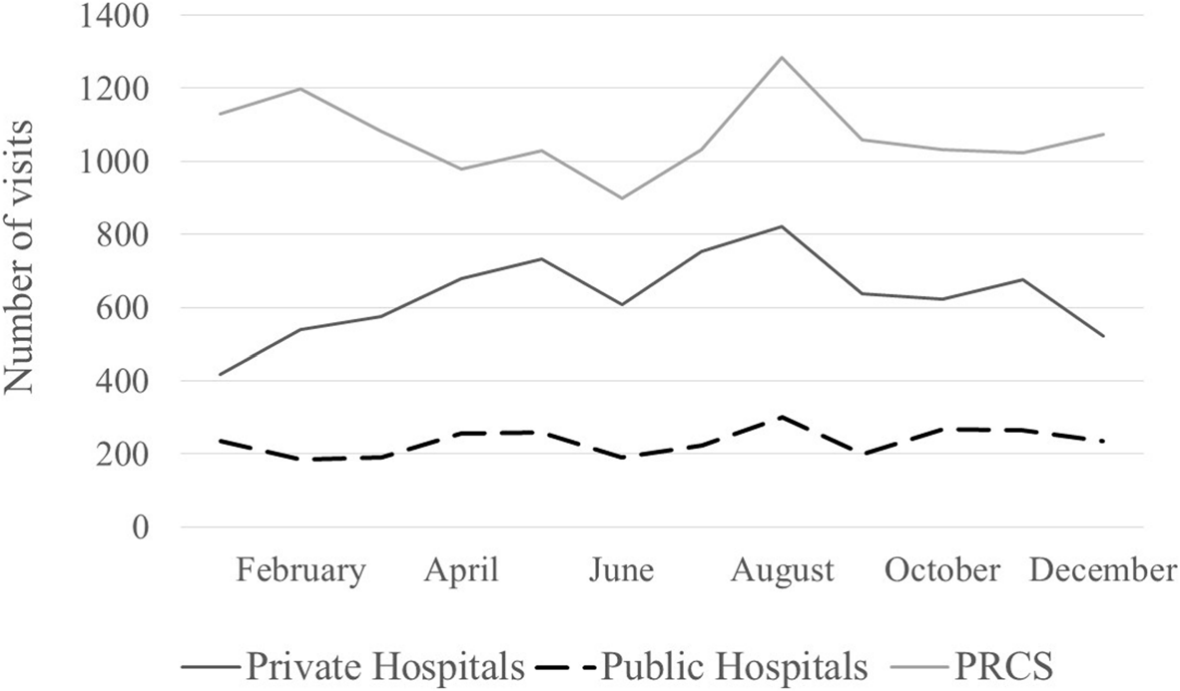
Average number of visits, per month, in 2016

A preliminary look into the data shows PRCS demand in terms of hospital visits was decreasing until June, when the second policy adjustment was put in place, as Fig. 2 shows. At the same time, demand for private hospitals seems to evolve in an opposite direction from that of PRCS. With the imposition of different cost-sharing levels in different hospitals, June was a turning point in terms of decision making for households with secondary health care needs.

A growing body of literature has examined the impact of cost-sharing policy implementation and abolition on health care demand. Nabyonga and co-authors present an impact assessment on the abolition of user fees in Uganda [9]. The authors carried out a longitudinal study in 106 health facilities across the country to explore how demand for health care services reacted to the policy change. The study found an increase in utilization among all population groups, with a relatively higher increase among the poor. Similar evidence was found by a study on the reduction of user fees for maternal care services [10]. The results of two multivariate logistic regression models suggested that poorer women might have benefited the most from the new financing policy with important implications for decreasing inequalities. Evidence from the Occupied Palestinian Territory also shows that out-of-pocket payments have a regressive effect and increase pre-existing income inequalities [11].However, in all the above cases user-fees (when known) were higher than the ones imposed by UNRWA in secondary hospitalization in 2016 and in most cases addressed a reduction rather than an increase in out-of-pocket fees.

Notably, one important tool that previous research has found to be effective in the successful implementation of new policies is to provide transparent and complete information to the community. This is especially true in complicated environments, where the population has few resources and is already struggling with day-to-day expenses. Indeed, studies have shown that a gradual introduction plan can be enough to transform a failed implementation into a smooth transition generally accepted by the population and with better results regarding budget saving outcomes [12–14].

The introduction of cost-sharing policies is a complex exercise as it has immediate negative implications for the user - costs increase. Nonetheless, some policies of this nature may actually bring important benefits to health care services [15, 16]. In UNRWA’s case, the new policy was introduced as a strategy adjustment for “greater sustainability and increased support for tertiary care", by shifting part of the coverage from secondary to tertiary level hospitalizations [17]. However, to what extent this policy was effective and what were the implied unforeseen effects is not clear. Of particular interest is whether users change behaviour after the cost-sharing policy is implemented and whether UNRWA is able to contain costs. Throughout this project, we answer these questions by analyzing how the bill value, UNRWA contribution and hospital visits change pre and post-intervention. For this purpose we focused on secondary care data for which we have pre and post policy information. This work is a valuable contribution towards increasing quality of health care for Palestine refugees, while providing a general framework of how hospitalization services are being used.

The overall findings suggest that introducing a 10% co-payment for secondary care for private and public hospitals had a significant impact redistributing demand between types of hospitals. Namely, after this policy change patients were at least 4 percentage points more likely to choose a PRCS hospital for secondary care. We could not find a defendable effect on the average number of stay in days, despite that UNRWA also improved occupancy control at the same time of the policy change.

The remainder of the study is organized as follows: Data set section presents the data and concerns of external validity, Methods section explains the methods used including the theoretical and empirical models, Results presents the main results and, finally, Conclusion provides conclusions and discussions of the analysis.

## Data set

The data used in this work are part of a broader ongoing program of data collection being conducted by UNRWA in all contracted hospitals with the goal of ultimately constructing a comprehensive time series of hospitalizations^1^.

For a matter of confidentially, the refugee registration number was anonymised, but in a way that allows to follow up of each patient. The data collection was initially piloted in 2013 and started being fully conducted in 2016.

**Fig. 3:**
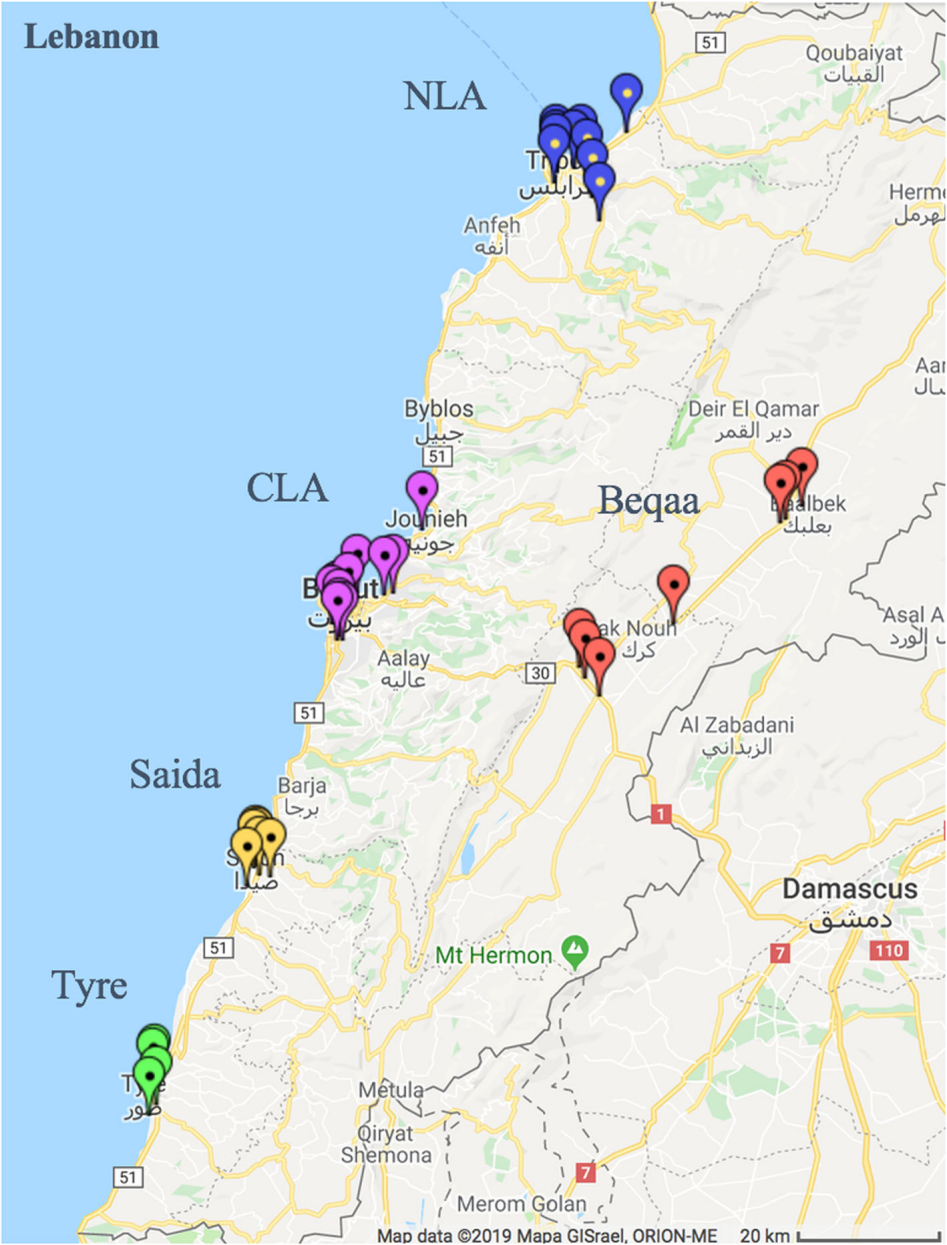
Hospitals location in Lebanon

For this project we use a subset of the original data from January 2016 to October 2017 with complete information on all UNRWA hospitalizations (the availability of the data depends on the on-going digitalization process). We focus on Palestine refugees from Lebanon in secondary care for which we have 32,061 observations, not including birth deliveries and MHF cases who benefit from a different financial support program and were differently affected by the policy change. We excluded MHF cases by eliminating observations from patients that got complete coverage using other than PRCS hospitals after June 2016 which is the best identification possible given that data on the MHF cases identifier is not available. The data contains individual level information collected from every hospital in UNRWA areas of operation, Beqaa, Central Lebanon Area (CLA), North Lebanon Area (NLA), Saida and Tyre, from 27 private hospitals, 5 PRCS and 4 public hospitals. The Lebanon map in Fig. 3 shows the distribution of UNRWA contracted hospitals across the country (more detailed maps by region in Additional file 1: Section A1.2). The available variables include the patients’ age and gender, entry and discharge date, diagnosis and surgery description, bill value, UNRWA contribution, patient contribution and hospitals’ characteristics. Because we have the complete population data-set, there is no need for sampling and the findings will be robust and representative of the population. This is an important strength of this paper that makes it unique in the literature.

**Fig. 4:**
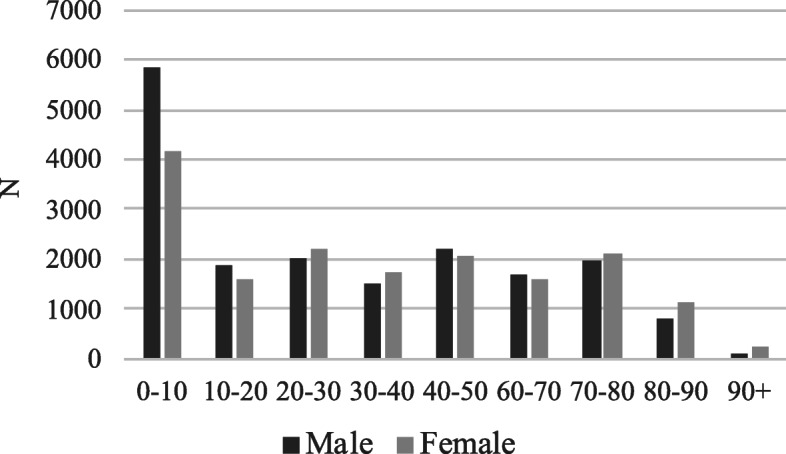
Population by age group and gender. Note: Data from January 2016 to October 2017 for secondary care

Gender of hospital users is generally equally distributed between males and females however, as seen in Fig. 4, there are some imbalances when data are stratified by age group. There is a relatively higher number of young males going to the hospital until the age of 10 to 20 years. Between 29 and 70 years, the groups are quite balanced and from 70 years onwards women become the majority. Existing evidence on Palestine youth shows that young males tend to have more dangerous behaviors that put their lives at risk, while females spend generally more time at home throughout their lives and end up living longer [18].

**Fig. 5:**
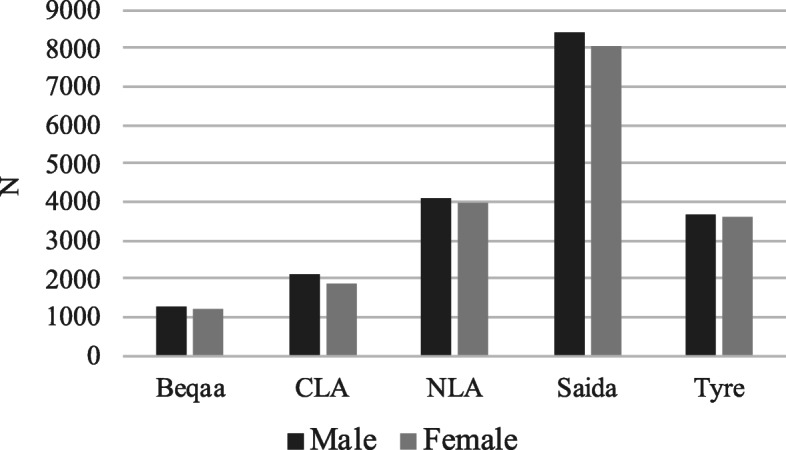
Population by region and gender. Note: Data from January 2016 to October 2017 for secondary care

In what concerns regional disparities, CLA is the area with the highest number of hospitals, 12, followed by NLA with 10, Beqaa with 7, Saida with 6 and Tyre with 4. Nonetheless, Saida has the highest number of incidents in the database, most likely due to having the highest population size and density of Palestine Refugees and Ein El Hilweh camp (in Saida), which were exposed to several conflicts during this period of time (Fig. 5).

Of the total observations 6,781 are surgical and 25,280 medical cases. Surgical cases are paid fee for service, independently of the number of days patients stay at the hospital. As such, these cases should not be affected by the higher monitoring from UNRWA at the time of the policy change. Regarding seasonality, the number of visits to the hospitals decreased significantly during Ramadan in both years and, especially because it coincided with the policy change in 2016, it is important for this to be considered in the analysis.

We are able to observe high responsiveness in the data with regards to the timings of policy changes and cultural events. This, combined with its representativeness, suggest a high quality and reliability of the data sources. The progress of the project was closely followed by UNRWA, that supervised and provided guidance on unregistered events and the general results interpretation related to culture and societal-specific features.

### External validity

The Palestine community living in Lebanon has a unique culture and has struggled with very particular social and political challenges over time. Although still registered as refugees, Palestine Refugees from Lebanon have been sharing the same geographic area as Lebanese for the last 70 years. On the other hand, this population continues to be marginalized and socially excluded with many living in precarious conditions. Moreover, with the Syrian refugee crisis, services became more crowded and scarcer in the country.

To assess how PRL compare with national averages we use the most recent data published, including the 2009 WHO Data Book for Lebanon, World bank data and the AUB Socieconomic Survey 2015 of Palestine refugees [8, 19].

Comparing AUB 2015 estimates for PRL in Lebanon, the Syrian Arab Republic and the West Bank and Gaza, we see generally a young population with approximately half under 24 years of age and West Bank and Gaza standing out with the highest percentage of population in this age group (Table 1) [8].

**Table 1:** Population in % of gender by age groups, 2015

	Lebanon		Syrian Arab Republic		West Bank and Gaza		PRL	
Age groups	Female	Male	Female	Male	Female	Male	Female	Male
0-24	46%	46%	52%	53%	61%	62%	45%	51%
25-64	48%	48%	44%	43%	36%	35%	46%	43%
65+	7%	6%	4%	4%	3%	3%	8%	7%

Poverty affects young Palestine refugees with 74% of adolescents living in poverty and 5% in extreme poverty, in line with recent evidence on the reality of other refugee groups such as Syrian [20]. The overall estimation is that 65% of PRL live below the poverty line, against 68% of Syrian refugees and 28.5*%* of Lebanese (UN Lebanon annual report 2018). PRL expenditures per month are also lower than the average of their Lebanese counterparts. Nonetheless, the employment rate for Lebanon was 43.9*%* slightly higher than the estimate for PRL of 37%, very close to the rate for the same year in Syrian Arab Republic and considerably higher than the 33.7*%* for West Bank and Gaza.

Regarding health indicators, the incidence of NCDs is high and increasing across the Arab world. The reported prevalence of chronic and acute disease among PRL is 37% and 63%, respectively. With heart disease, stroke and diabetes as the top three causes of death in Lebanon, the most common NCDs are similar for both groups and a common issue across the region [21, 22]. Infant mortality rates on the other hand, show slightly lower values for PRL at 19 per 1000 births, compared to 21 in Lebanon, 29.6 in Syrian Arab Republic, 21 in West Bank and 23 in Gaza. This is an indicator that is usually strongly correlated with life expectancy [23]. In this sense, these indicators highlight that the PRL population in Lebanon have strong similarities with other countries and refugee populations across the region. This is one of the key factors strengthening external validity and making this policy impact analysis valid in similar contexts.

## Methods

### Theoretical model

To understand patient behavior following the introduction of a co-payment in secondary care hospitalization costs, we develop a theoretical model that formalizes a hypothesis on how individuals decide between hospitals^2^.

We are studying the policy implementation as a natural experiment in two stages, the ex ante stage where patients have free access to secondary care, and the ex post stage where treatment in public and private hospitals is charged at 10%, but not in PRCS hospitals. Given this setting, we assume the different hospital types have different quality levels and use a vertical differentiation model to analyze competition and interaction among hospitals. Providers compete in terms of quality, which is valuable because it can result in better health outcomes or improve the treatment process itself.

We start by considering that patients obtain utility from their treatment. This utility is directly influenced by the cost and benefit of treatment, which in turn are dependent on the condition’s severity level. In this market for health care there are two hospitals, indexed by j=1,2, where hospital 1 is of higher quality than hospital 2 and patients have preferences for these hospitals. The treatment cost share is exogenous and can vary over time and between hospital type. The demand each provider faces is then determined by the preference of the indifferent patient.

Each patient makes the decision to take treatment or not and from which hospital to demand treatment. This said, the patients’ utility *U*(*j*,*η*),*j*=1,2, with disease severity *η*, when choosing provider *i* is given by:

1 $$ U(j,\eta)=\theta_{j} B(\eta) - S_{j}C_{j}(\eta)  $$

where *B*(*η*) is the benefit of getting treatment, which we assume to be equal for all patients of the same severity whatever hospital they select, and *S*_*j*_ is the share of the total cost, *C*(*η*) (measured in USD units)^3^, that the patient is required to pay, i.e., *S*_*j*_*C*_*j*_(*η*) is the out-of-pocket payment, exogenously established. Both *C*_*j*_(*η*) and *B*(*η*) are increasing on severity (*B*^′^(*η*)>0,*B*^″^(*η*)<0,*Cj*′(*η*)>0,*Cj*″(*η*)>0), meaning that higher severity corresponds to higher benefits, but also higher cost. The augmented preference for the quality hospital is given by *θ*, where *θ*_1_>1 and *θ*_2_=1, such that the benefit of getting treatment at hospitals with higher quality is larger. In this framework, consider that:

- *η*_1_>*η*_2_⇒*C*_*j*_(*η*_1_)>*C*_*j*_(*η*_2_),∀*η*;
- *S*_1_>*S*_2_; and
- *C*_1_(*η*)>*C*_2_(*η*),∀*η*;

Then:

1. There exists a *η*^∗^ such that, for *η*≥*η*^∗^:
  *B*(*η*)≥*S*_2_*C*_2_(*η*), everyone gets treatment at the hospital;
2. There exists a *η*^∗∗^ such that,
  For *η*^∗^<*η*<*η*^∗∗^:
  *θB*(*η*)−*S*_1_*C*_1_(*η*)<*B*(*η*)−*S*_2_*C*_2_(*η*), everyone chooses hospital 2.
  And for *η*≥*η*^∗∗^:
  *θB*(*η*)−*S*_1_*C*_1_(*η*)>*B*(*η*)−*S*_2_*C*_2_(*η*), everyone chooses hospital 1.

To study these conditions we need to understand how the thresholds vary with changes in out-of-pocket payments, *S*_*j*_.

For this purpose, we derive the severity thresholds functions, *η*^∗^ and *η*^∗∗^, in order to *S*_*j*_, through the application of the Implicit Function Theorem:

If f : *R*^*m*^×*R*⇒*R* is a *C*^1^ function, *f*(*x*_0_;*y*_0_)=0, and $\frac {\partial f}{\partial x} \neq 0$, then for some neighborhood *U*⊂*R*^*m*^ of (*x*_0_) there is a *C*^1^ function *g*:*U*⇒*R* such that *g*(*x*_0_)=*y*_0_ and *f*(*x*,*g*(*x*))=0 for all *x*∈*U*. The partial derivatives of *g* at *x*_0_ are given by the formula:

$$ \begin{aligned}[b] \frac{\partial g}{\partial x^{i}}(x) = - \frac{\frac{\partial f}{\partial x^{i}}(x_{0},y_{0})} {\frac{\partial f}{\partial y}(x_{0},y_{0})} \end{aligned} $$

The calculations yield the following results (proofs in Additional file 1: Section A1.1):

#### **Proposition 1**

The severity threshold for patients to get treatment, *η*^∗^, is positively related to treatment costs at the low quality hospital, *S*_2_*C*_2_(*η*). This is, as the patients’ contribution share increases, *S*_2_, the severity threshold that leads a patient to seek treatment increases.

2 $$ \begin{aligned}[b] \frac{\partial \eta^{*}}{\partial S_{2}}(S_{2})= \frac{C_{2}(\eta^{*})}{\frac{\partial B(\eta^{*})}{\partial \eta^{*}} - S_{2}\left(\frac{\partial C_{2}(\eta^{*})}{\partial \eta^{*}}\right)} >0 \end{aligned}  $$

#### **Proposition 2**

An increase in patient contribution charged in hospital 1, *S*_1_, increases the severity threshold that leads patients to change their choice to the high quality hospital, *η*^∗∗^.

3 $$ \begin{aligned} \frac{\partial \eta^{**}}{\partial S_{1}}(S_{1}, S_{2})= \frac{C_{1}(\eta^{*})}{\frac{\partial B(\eta^{**})}{\partial \eta^{**}} - S_{1}\left(\frac{\partial C_{1}(\eta^{**})}{\partial \eta^{**}}\right)} > 0 \end{aligned}  $$

#### **Proposition 3**

An increase in patient contribution charged in hospital 2, *S*_2_, decreases the severity threshold that leads patients to choose the high quality hospital, *η*^∗∗^.

4 $$ \begin{aligned} \frac{\partial \eta^{**}}{\partial S_{2}}(S_{1}, S_{2})= - \frac{C_{2}(\eta^{**})}{\frac{\partial B(\eta^{**})}{\partial \eta^{**}} + S_{2}\left(\frac{\partial C_{2}(\eta^{**})}{\partial \eta^{**}}\right)} < 0 \\ \end{aligned}  $$

#### **Proposition 4**

An increase (decrease) in patient contribution charged in hospital 1, *S*_1_, decreases (increases) the number of patients going to hospital 1 and increases (decreases) the number of patients going to hospital 2.

5 $$ \begin{aligned} \frac{\partial W_{1}}{\partial S_{1}} < 0 ;\frac{\partial W_{1}}{\partial S_{2}} > 0; \ \ \frac{\partial W_{2}}{\partial S_{1}} > 0;\frac{\partial W_{2}}{\partial S_{2}} < 0 \end{aligned}  $$

Where, *W*_*j*_ is the number of patients going to each hospital. Since from Proposition 3 the severity threshold of going to hospital 1 increases, the average costs and length of stay are also expected to increase at hospital 1.

**Fig. 6:**
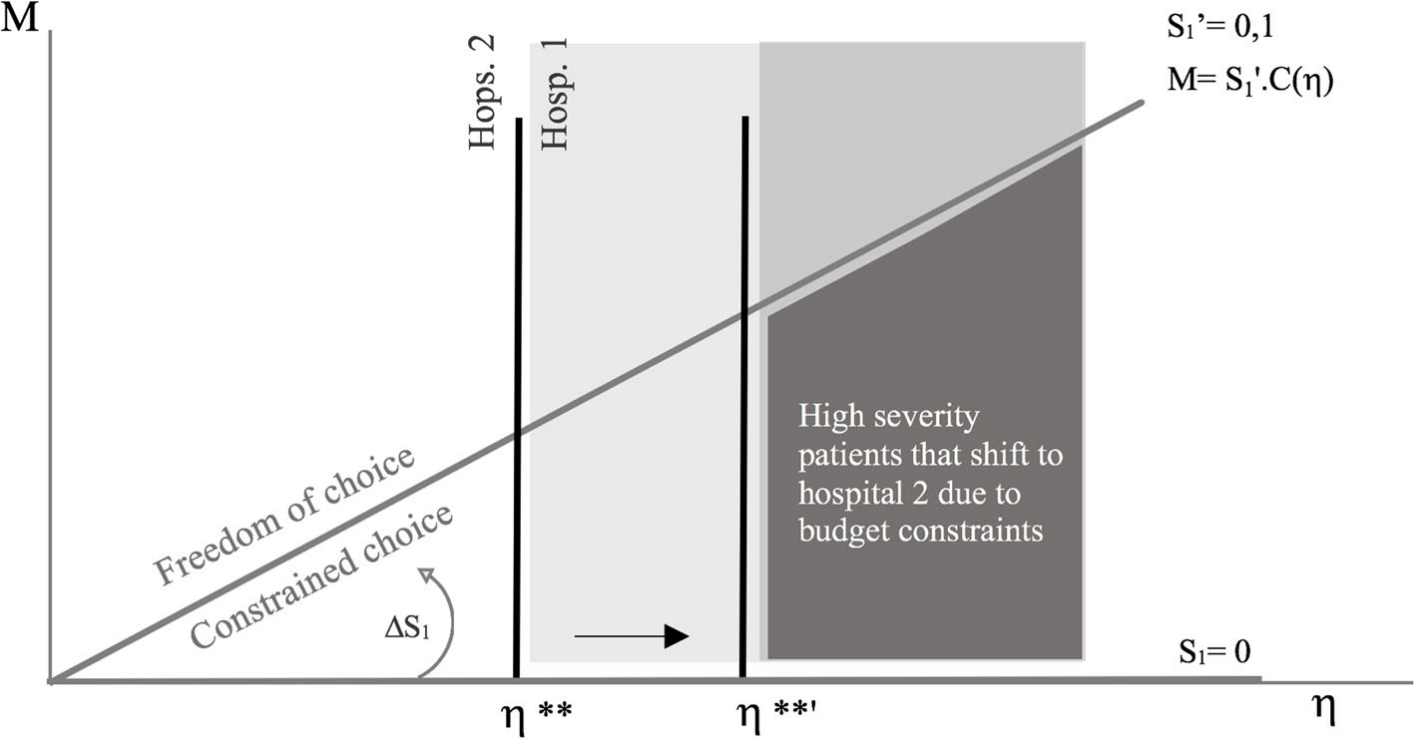
Budget constraint dynamics (from *S*_1_=0 to $S_{1}^{'}=0,1$)

Additionally, because this is a context of strong financial distress, it is important to consider that some heavy users with severe cases at high quality facilities will be forced to shift hospital due to lack of financial resources [8]. In order to include these cases in the model we need to add a budget constraint at the patient level, such that:

- *M*>*SC*(*η*_1_);

To understand further how this condition interferes with the model, Fig. 6 shows how the increase in patient contribution in hospital 1 from 0 to 10% can affect their choices.

Consider the budget constraint and the increase on patient contribution, *S*_1_. From the above explained theory, there is a threshold severity level *η*^∗∗^ after which patients will prefer to choose a high quality hospital (hospital 1). We also saw that when *S*_1_ increases, the severity threshold, *η*^∗∗^, increases (from *η*^∗∗^ to *η*^∗∗^^′^ in the graph) and it takes a more severe health condition to make people willing to pay more. Following, the number of patients decreases and the average patient length of stay (LoS) increases at hospital 1. However, from the group of people that are willing to pay more, some will not be able to follow this increase in costs. Taking this into account, more people will shift to hospital 2, and there will be a negative effect on the average LoS at hospital 1. The overall effect on average LoS at hospital 1 will be a trade-off between the two effects described. In Fig. 6, the light grey area represents the point after which patients choose hospital 1 and the dark grey represents those that are willing to pay, but will choose a low quality hospital because *M*<*SC*(*η*_1_).

Applying this theoretical reasoning to the study, we can consider PRCS hospitals to be hospital type 2 and private and public hospitals to be hospital type 1. We will use the relationships above to interpret the results achieved from the econometric results, in order to understand the rational behind the patients’ behavior changes. Let us consider two hypothesis, following the introduction of a 10% co-payment as of June 2016:

- Patients that shift to hospital 2 due to financial constraints were overusing the high quality hospital, when the low quality hospital has enough resources to treat all diseases and conditions with lower costs for UNRWA;
- Patients that shift to hospital 2 due to financial constraints will not have access to sufficient care and this will have negative future impacts in terms of level of morbidity and mortality.

If the first hypothesis is confirmed, the new policy was effective to reduce inefficiencies and allowed UNRWA to contain costs, on the other side, the second hypothesis implies that the policy not only did not allow UNRWA to contain costs, but also made access to healthcare more difficult for poorer families with severe health conditions. The estimation and econometric methods adopted will allow us to explore these hypotheses and understand what is the most plausible scenario according to the data under analysis.

### Estimation and econometric methods

The main purpose of this study is to estimate the effect of co-payments on patients’ healthcare decisions - PRL - and the providers costs - UNRWA. We exclude the period between January and March 2016 and focus on the shift from full coverage (in force during April and May 2016) to cost-sharing (after June 2016). We use a differences estimation equation, as follows:

6 $$  Y_{it}= \alpha + \beta_{1}T + \beta_{2}X_{it} + \beta_{3}H_{it} + \epsilon_{it}  $$

Where *i* = 1,.., N denotes individuals and t represents time (day).

The dependent variable, *Y*_*it*_, corresponds to each outcome of interest (all monetary variables will be expressed in USD):

- Categorical variable for hospital type with 3 categories: choosing a PRCS, a public or a private hospital - Multinomial logit model;
- Count variable for stay in days - Negative binomial model;
- Continuous variables for patient contribution, UNRWA contribution and bill value - one linear regression for each.

Note that for the main estimation the dependent variables will not be included in each other estimations to avoid endogeneity issues affecting the results. Nonetheless, in the robustness checks in Additional file 1: Sections A1.3.1 and A1.3.2, for example, in the demand specification, stay in days and bill value are included as controls.

T is a treatment vector time-varying independent variable (policy variable in The policy variable section) with coefficient *β*_1_, such that T=1 if the period is after the last policy change (from June 2016 onward) and T=0 otherwise. Matrix *X*_*it*_ includes individual-specific characteristics, including gender and age, *H*_*it*_ corresponds to the demographic profile and characteristics of the hospitals (region, distance to refugee camp, type), and *ε*_*it*_ is the error term (all estimations were applied in Stata 14.0 with support from Microsoft Office Excel 2016). In simple differences analysis the event under study must be exogenous to the outcome variables, which is verified in this case as the policy change was an exogenous decision taken by UNRWA.

We use a total population, where the individuals are observed each time they use hospitalization services (i.e. hospital inpatients) and it is thus not necessary to use fixed effects methods for the results to be robust^4^. Nonetheless, to avoid heterogeneity issues we use clustered standard errors by hospital in all estimations and robust standard errors for further robustness checks presented in Additional file 1: Section A1.3.

Because we wish to identify mechanisms through which the policy change had an impact on several features of hospitalization services we estimate various specifications of the general model in (6), with different estimation methods, depending on the dependent variables.

#### Dependent variables

The policy change under analysis implied different coverage between hospitals that may have had an impact on the patients’s choice. With a complete database of hospitalization cases, every individual episode corresponds to one out of the three hospital types - private, public or PRCS. In this framework, we conducted a multinomial logit model, where the outcome variable is hospital type, a categorical with values from 1 to 3, where 1 corresponds to PRCS, 2 to private and 3 to public hospitals. Following the theoretical reasoning in the previous section, this approach assesses the indirect utility of each alternative, assuming that individuals choose the one that provides the greatest utility [24]. The dependent variable is thus the indirect utility of each choice as a function of individual, hospital and unobserved characteristics. The coefficient estimates give the differential effects of the observed characteristics on utility, from which we compute the average marginal effect of each variable.

The second estimation exercise focuses on measuring the policy impact on the Length of stay (LoS) at UNRWA contracted hospitals for secondary care. LoS is calculated as the number of days between the admission and the discharge date of a given patient and can be considered as a severity indicator in the sense that more severe conditions are associated with longer hospitalization periods. In addition, simultaneously with the policy change, UNRWA also increased LoS monitoring for all patients covered by UNRWA. If this measure was efficient, LoS is expected to decrease in all hospitals, potentially decreasing also bill values for medical cases. To perform this estimation we use a negative binomial regression model, largely used for non-negative integer dependent variables with over-dispersion (variance is more than double of the mean), as it is the case [27, 28]. As a robustness check we also perform the same regression using multilevel poisson estimation model, presented in Additional file 1: Section A1.3.2.

To understand the financial consequences of these changes we turn our focus to the impact on bill value, UNRWA and patient contribution. The bill value corresponds to the total costs health-care by individual, including the procedure’s value, doctors services payment, occupancy and medication expenditures (while hospitalized) or a fixed fee in case of surgery. This value is then presented to the patient, who receives financial support from UNRWA that usually corresponds to a fixed share of the total bill value (in secondary care, the sum between UNRWA and patient contribution is generally equal to the total bill value, with some exceptions for when the patient receives support from a third contributor). These three continuous variables present a left-skewed distribution which is common in health-care costs data due to the presence of few heavy users [29]. In order to properly use the Ordinary Least Squares (OLS) model we performed logarithm transformations so that we can use normally distributed variables. For this set of estimations, in Eq. 6*Y*_*it*_ becomes *log*(*Y*_*it*_). Although this implies a loss of accuracy, this method is widely in used in the literature for these situations and studies have proven its robustness [30].

#### The policy variable

For the main specification of the hospital demand estimation model, the policy variable is binary with the value 0 for the period that preceded the policy change and the value 1 for the period after the new co-payment policy was implemented. This means that the variable ‘policy’ has the value zero until June 2016 and value one from that moment onward. This variable is our main object of study to identify the main changes in healthcare demand after UNRWA introduced a 10% co-payment on hospital care for secondary level at all but PRCS hospitals.

As explained in UNRWA hospitalization policy changes: a natural experiment section, the first version of this policy happened between January and March 2016 and was introduced as a co-payment between 5% and 20% (depending on the hospital). This scheme was discontinued and there was a period of negotiation when care was free of charge again. The last version was finally agreed and implemented in June 2016. To estimate the main policy impact we focused on the last and more permanent change, without taking into account the first three months of 2016 when the first version was in place. With this specification we are comparing a free-of-charge treatment with a 10% co-payment directly. This gives us a specific moment in time associated to a specific change, which is effectively a regression discontinuity design for a single arm, due to an external shock. On the other side, this setting implied that the pre-policy data was just around 12% of the whole sample, creating some potential identification issues.

Using data from April to July 2016, we conduct an estimation where pre and post policy data both have the length of 2 months (total of 4 months) to improve the identification strategy, maintaining the policy variable unchanged. Using data from January to October 2016, with pre and post policy data length of 5 months (total of 10 months) we estimate the impact on PRCS demand with two policy variables, one for the first and one for the second version of the policy. In this setting, the first policy variable gets the value 1 from January to March 2016, and 0 for the remaining of the timeline, and the second (same as before), with the value 0 from January to May 2016 and the value 1 from June 2016 onward. The binary policy variables here indicate periods when treatment was not for free, compared to the periods that it was. We then do this same specification for the whole sample, January 2016 to October 2017, and again, but with monthly fixed effects.

Table 2 shows the descriptive statistics for the main variables of interest, by hospital type at the different stages of the policy.

**Table 2:** Descriptive statistics, by hospital type and policy stage

	Policy Jan-Mar	Policy Apr.-May	Policy Jun +
Percentage of total patient			
PRCS	62%	51%	55%
Private	27%	36%	33%
Public	11%	13%	12%
Bill value (USD)			
PRCS	186.19	186.30	183.57
Private	531.33	470.39	512.91
Public	544.01	470.17	483.27
Patient contribution (USD)			
PRCS	9.10	3.37	1.85
Private	116.82	24.51	80.70
Public	75.91	10.46	56.95
Age			
PRCS	35.59	35.22	34.93
Private	38.12	36.89	36.87
Public	32.01	30.36	31.29
Length of Stay (days)			
PRCS	2.05	1.95	1.97
Private	2.96	2.58	2.74
Public	3.42	3.04	3.03

All methods were performed in accordance with the relevant guidelines and regulations.

## Results

Considering the theoretical framework presented, the empirical results explore whether patients are using services more efficiently after the introduction of 10% co-payment costs for certain hospital types, or whether access to hospital services became more difficult for families with severe health conditions and in financial distress. The estimation results in this section will help us achieve the answers. Table 3 presents a summary of our main results that we will describe in detail throughout this section.

**Table 3:** Main estimations - Summary table

	Mult. Logit - Margins	Neg. Bin. - IRR	OLS		
	PRCS	LoS	Bill value	UNRWA contr.	Patient contr.
	(1)	(2)	(3)	(4)	(5)
Policy	0.035***	0.995	-0.030	-0.060**	-0.038
	(0.009)	(0.026)	(0.019)	(0.024)	(0.205)
Age	0.002***	0.987***	-0.009***	-0.009***	-0.009***
	(0.000)	(0.003)	(0.002)	(0.002)	(0.002)
Age^2^	-0.000***	1.000***	0.000***	0.000***	0.000***
	(0.000)	(0.000)	(0.000)	(0.000)	(0.000)
Woman	0.007	0.967**	-0.035***	-0.032***	-0.071***
	(0.006)	(0.013)	(0.011)	(0.011)	(0.025)
Ramadan	-0.013	0.981	-0.022	-0.028*	0.010
	(0.010)	(0.022)	(0.018)	(0.015)	(0.066)
Distance	0.031***	1.014*	0.009	0.010	0.061*
	(0.001)	(0.009)	(0.013)	(0.011)	(0.031)
Visit	-0.001	1.028***	0.024***	0.023***	0.048***
	(0.002)	(0.009)	(0.006)	(0.005)	(0.010)
Surgery	0.029***	0.714***	0.461***	0.452***	0.701***
	(0.007)	(0.062)	(0.080)	(0.080)	(0.106)
CLA	0.402***				
	(0.015)				
Region FE		Yes	Yes	Yes	Yes
Constant		1.947***	12.320***	4.976***	2.647***
		(0.256)	(0.187)	(0.166)	(0.207)
Observations		33,469	33,402	33,401	13,134
R-squared			0.128	0.133	0.233

^1^*** p<0.01, ** p<0.05, * p<0.1. Standard errors clustered by hospital in parentheses

Table 4 shows the results for the multinomial logit model that measures the impact of each explanatory variable on the probability of going to each hospital type. The policy coefficient had a positive and statistically significant impact on the probability of an episode happening at a PRCS (demand) and the opposite effect for Private hospitals. Namely, after June 2016 patients were around 4 percentage points (pp) (p <0.01) more likely to choose a PRCS hospital instead of a private or public hospital (note that because it is a multinomial logit, patients are distributed across the three hospital types, as such when the demand changes for one of them it has to fully compensate in at least one of the others). Regarding public hospitals, the database includes 17,287 observations for PRCS, 7,208 for private and 2,679 for public hospitals. Since this is a complete population dataset and there are significantly fewer public hospitals, demand for these hospitals will most likely be driven by particular reasons such as distance. As robustness check we added bill value, UNRWA and patient contribution and length of stay to the explanatory variables (Additional file 1: Section A1.3). Those results show a positive and statistically significant impact of 18 pp on demand for PRCS. While the effect of the policy itself might be larger than the one found in our main result, because the added variables can be endogenous to the outcome, we cannot be completely certain they are not causing a bias in the results.

**Table 4:** Policy impact estimation on demand for hospital type (Multinomial logit - margins), from April 2016 to October 2017

	(1)		(2)		(3)	
	PRCS		Private		Public	
Policy	0.035***	0.037***	-0.019**	-0.017	-0.016***	-0.019**
	(0.009)	(0.012)	(0.008)	(0.011)	(0.005)	(0.008)
Age	0.002***	0.002***	0.000	0.000	-0.003***	-0.003***
	(0.000)	(0.000)	(0.000)	(0.000)	(0.000)	(0.000)
Age^2^	-0.000***	-0.000***	0.000	0.000*	0.000***	0.000***
	(0.000)	(0.000)	(0.000)	(0.000)	(0.000)	(0.000)
Woman	0.007	0.007	-0.005	-0.005	-0.003	-0.003
	(0.006)	(0.006)	(0.005)	(0.005)	(0.003)	(0.003)
Ramadan	-0.013	0.001	0.010	-0.011	0.003	0.010
	(0.010)	(0.017)	(0.009)	(0.015)	(0.006)	(0.011)
Distance	0.031***	0.031***	0.021***	0.021***	-0.052***	-0.052***
	(0.001)	(0.001)	(0.001)	(0.001)	(0.002)	(0.002)
Visit	-0.001	-0.000	-0.002	-0.002	0.002***	0.002**
	(0.002)	(0.002)	(0.001)	(0.001)	(0.001)	(0.001)
Surgery	0.029***	0.029***	-0.007	-0.007	-0.022***	-0.022***
	(0.007)	(0.007)	(0.006)	(0.006)	(0.005)	(0.005)
CLA	0.402***	0.402***	-0.446***	-0.446***	0.044***	0.043***
	(0.015)	(0.015)	(0.017)	(0.017)	(0.007)	(0.007)
Month FE		Yes		Yes		Yes
Observations	33,469	33,469	33,469	33,469	33,469	33,469
Demand^3^ (%)	55.43		33.14		11.43	

^1^*** p<0.01, ** p<0.05, * p<0.1. Robust standard errors in parentheses

^2^Note: The dependent variables are binary variables with the value 1 if the patient is at each hospital type and 0 otherwise. Note that all patients get treatment, thus for each observation at least one option must be selected. Coefficients show average marginal effects for multinomial logit regression results. Policy is a dummy variable that indicates the period after the last policy change (from June 2016 onwards). These model specifications control for individual and hospital specific variables

^3^Share of total visits by hospital type using full sample

Apart from the general demand shift, there is also a noteworthy preference of surgeries being performed at PRCS hospitals which can be a further indicator that costs matter for this population. Surgeries in this sample are more expensive on average in every hospital type, which can lead patients to chose the cheaper option.

Because we are dealing with limited pre-policy data and a long post policy period, Table 5 shows results for the hospital demand estimation (PRCS outcome), but using different time intervals. We estimate the model with only 2 months pre and post policy, with 5 months pre and post policy and two policy dummies, for the full sample (January 2016 to October 2017) and the full sample with monthly fixed effects. With all specifications the policy impact is similar as before, going from a not significant result with only 2 months previous and post policy implementation to an increase of 3.7 pp (p <0.01) in the probability of visiting a PRCS hospital with full sample and monthly fixed effects,

**Table 5:** Policy impact estimation on demand for PRCS hospitals (Multinomial logit - margins), different periods

	PRCS demand			
	(1)	(2)	(3)	(4)
Policy - Jan		0.112***	0.112***	0.109***
		(0.020)	(0.020)	(0.016)
Policy - Jun	0.011	0.034**	0.035**	0.037***
	(0.014)	(0.017)	(0.015)	(0.012)
Age	0.003***	0.003	0.002	0.002***
	(0.001)	(0.002)	(0.002)	(0.000)
Age^2^	-0.000***	-0.000*	-0.000*	-0.000***
	(0.000)	(0.000)	(0.000)	(0.000)
Woman	0.017	0.023***	0.010*	0.010*
	(0.012)	(0.006)	(0.006)	(0.005)
Ramadan	0.020	-0.006	-0.013	0.001
	(0.018)	(0.014)	(0.012)	(0.017)
Distance	0.036***	0.035	0.031	0.031***
	(0.003)	(0.059)	(0.059)	(0.001)
CLA	0.489***	0.452	0.406	0.406***
	(0.040)	(0.341)	(0.328)	(0.014)
Visit	-0.007	0.001	-0.001	-0.000
	(0.007)	(0.010)	(0.006)	(0.002)
Surgery	0.055***	0.038	0.031	0.031***
	(0.015)	(0.035)	(0.034)	(0.007)
Month FE				Yes
Observations	6,961	17,621	38,562	38,562

^1^*** p<0.01, ** p<0.05, * p<0.1. Robust standard errors in parentheses

^2^(1) 2 months pre and post policy

(2) 5 months pre and post policy

^1^(3) Full sample - January 2016 to October 2017

^1^(4) Full sample with month FE - January 2016 to October 2017

^3^Note: The dependent variables are binary variables with the value 1 if the patient is at each hospital type and 0 otherwise. Note that all patients get treatment, thus for each observation at least one option must be selected. Coefficients show average marginal effects for multinomial logit regression results. Policy is a dummy variable that indicates the period after the last policy change (from June 2016 onwards). These model specifications control for individual and hospital specific variables

If it is the case that PRLs were more likely to choose PRCS hospitals after the policy change, we should be able to confirm this by looking at the impact on frequency of visits. To do this, first, we collapsed the data to the week level and normalized the hospital volume by the estimated number of PRLs at the time (280,000 [31]). Second, we conducted a linear regression on the normalized variable that gives the number of hospital visits per 1,000 PRLs, as a function of the visit being at a PRCS hospital, before and after the different policies, and some characteristics also used in the other regressions. Note that we used specification [4] from the last estimation, with the full sample, two policy variables, and monthly fixed effects, because collapsing the data reduces the number of observations dramatically. This estimation also lets us study the effect on the extensive margin, i.e., if people continued to use hospitals at the same rate as before the co-payment was implemented.

Table 6 presents the results for the linear coefficients. We find that, as before, both policies have a negative general impact on visits, but a positive impact on visits at PRCS, given by the interaction terms between the policy binary variables and going to a PRCS. Going to a PRCS hospital after the 10% co-payment was implemented is associated with 0.28 more visits per 1,000 PRL (p <0.05). As for the extensive margin, after the last policy was implemented the number of visits per 1,000 PRL decreased in 0.29 (p <0.05). The impact of the first policy (Policy Jan) has the same direction as the one for the policy change implemented in June 2016 (Policy Jun), but with a larger magnitude and a more statistically significant coefficient. This result was expected since the first policy was harsher and less predictable than the last one.

**Table 6:** Policy impact on the demand volume of visits, by week (OLS)

	Freq by 1000 PRL
	(1)
PRCS	0.533***
	(0.143)
Policy - Jan	-0.539***
	(0.127)
Policy Jan x PRCS	0.537***
	(0.189)
Policy - Jun	-0.285**
	(0.118)
Policy Jun x PRCS	0.280*
	(0.155)
Age	0.000
	(0.002)
Distance	0.277***
	(0.026)
Ramadan	-0.028
	(0.112)
CLA	-1.288***
	(0.043)
Constant	0.190
	(0.157)
Observations	632
R-squared	0.732

^1^*** p<0.01, ** p<0.05, * p<0.1. Robust standard errors in parentheses

^2^Full sample with month FE (January 2016 to October 2017)

^3^Note: We collapsed data to week level and normalized the hospital volume by the estimated number of PRLs in 2016 (280,000). The outcome variable is the number of hospital visits per 1,000 PRLs. Policy Jan is a dummy variable that indicates the period after the first policy change (from January to March 2016 onwards); and Policy Jun indicates the last version implemented in June 2016

These results make us more confident of the potential impact of co-payments on healthcare demand.

Looking at the policy impact on LoS, Table 7, in the main estimation we found no statistically significant effect after the co-payment was implemented. Since UNRWA increased monitoring at the same time the policy was implemented, it can be that both effects balanced off or that none had an impact. In particular, in PRCS the change in control and the introduction of a co-payment have two opposite effects. On one side, average stays should be shorter due to the increase in control, on the other, LoS is expected to increase with demand, especially if that demand shift is driven more by users with more severe conditions. Going back to the table, the interaction coefficients between hospital types and the policy are above 1 (relative to PRCS). This means, the average number of days at the hospital per episode was higher at these hospitals after the policy being implemented, but the impact is not statistically significant.

**Table 7:** Policy impact estimation on Stay in Days (Neg. Binomial - IRR), from April 2016 to October 2017 (with controls)

	Length of Stay		
	(1)	(2)	(3)
Policy	0.995	0.988	0.990
	(0.026)	(0.025)	(0.038)
Private hospital		1.441***	1.441***
		(0.132)	(0.132)
Public hospital		1.423***	1.423***
		(0.188)	(0.189)
Public hospital x Policy		1.037	1.037
		(0.057)	(0.057)
Private hospital x Policy		1.023	1.023
		(0.122)	(0.123)
Age	0.987***	0.988***	0.988***
	(0.003)	(0.003)	(0.003)
Age^2^	1.000***	1.000***	1.000***
	(0.000)	(0.000)	(0.000)
Woman	0.967**	0.971**	0.971**
	(0.013)	(0.012)	(0.012)
Ramadan	0.981	0.974	0.926
	(0.022)	(0.022)	(0.053)
Distance	1.014*	1.018***	1.018***
	(0.009)	(0.004)	(0.004)
Visit	1.028***	1.026***	1.027***
	(0.009)	(0.009)	(0.009)
Surgery	0.714***	0.726***	0.726***
	(0.062)	(0.064)	(0.064)
Region FE	Yes	Yes	Yes
Month FE			Yes
Constant	1.947***	1.440***	1.470***
	(0.256)	(0.133)	(0.165)
Observations	33,469	33,469	33,469
Mean value (days)	2.362		

^1^*** p<0.01, ** p<0.05, * p<0.1. Robust standard errors in parentheses

^2^Note: Coefficients show Incidence Rate Ratios (IRR) for a negative binomial regression results. Standard errors clustered by hospital in parentheses. Policy is a dummy variable that indicates the period after the last policy change (from June 2016 onward). This model specification controls for individual and hospital specific variables

Because LoS distribution is highly skewed to the right and most patients in this sample stay only one day at the hospital, we divide the sample into two groups: the ones that stay one day at the hospital and the ones that stay at least two (there are only 4 observations that stay less than one day at the hospital and they were excluded for this part of the analysis). Following the results in Table 8, with this specification, after the policy was implemented the probability of going to a PRCS hospital was lower among episodes with shorter stays. Considering the main result that the general demand increased, if the policy had a negative impact on shorter stays, patients with quicker treatments were less attracted to the cheaper option. This can be because of an overcrowding effect of people with longer stays, despite the results for the later not being significant. This follows the theoretical hypothesis that the policy change was more significant impact for those which the 10% meant a more significant cost (i.e. higher bill values and longer stays).

**Table 8:** Policy impact estimation on PRCS demand by LoS (Neg. Binomial - IRR), from April 2016 to October 2017 (with controls)

	PRCS	
	(1)	(2)
	1 day	2+ days
Policy	-0.058**	-0.020
	(0.025)	(0.013)
Age	0.001	0.002
	(0.002)	(0.002)
Age2̂	-0.000	-0.000
	(0.000)	(0.000)
Woman	0.021	0.005
	(0.013)	(0.008)
Ramadan	0.002	-0.036***
	(0.019)	(0.011)
Distance	0.022	0.038
	(0.054)	(0.060)
Visit	0.000	-0.003
	(0.010)	(0.007)
Surgery	-0.190***	0.290***
	(0.057)	(0.039)
CLA	0.408	0.373
	(0.334)	(0.309)
Observations	14,706	23,852

^1^*** p<0.01, ** p<0.05, * p<0.1. Robust standard errors in parentheses

^2^Note: Dependent variables in log transformations; Estimations include controls for type of hospital, gender, age, Ramadan and LoS. Standard errors clustered by hospital in parentheses. Policy is a dummy variable that indicates the period after the last policy change (from June 2016 onwards)

In what concerns costs, Table 9 shows that the policy change had an impact on UNRWA contribution, which reflects the policy itself. This effect naturally disappears when we control for hospital type, since it was not implemented in all types. We do find negative and statistically significant results on the interactions between the policy and hospital type for patient contribution, which goes in line with the main evidence that demand for PRCS increased. Overall, we could not find any other significant impact on any of the three outcome variables - bill value, UNRWA and patient contribution. If on one side, UNRWA provided less financial support for patients going to more expensive hospitals, on the other, more people are going to the cheapest option (PRCS). If both effects balance out than the impact on costs is expected to be diminished and potentially not significant. In other words, demand reacted to the policy change by changing their hospital choice, which might have been enough to accommodate the changes in costs.

**Table 9:** Policy impact estimation on Bill value, UNRWA contribution and Patient contribution (OLS), from April 2016 to October 2017 (with controls and all dependent variables in logarithm)

	Bill value		UNRWA contribution		Patient contribution	
	(1)	(2)	(3)	(4)	(5)	(6)
Policy	-0.030	-0.044	-0.060**	-0.035	-0.038	0.092
	(0.019)	(0.028)	(0.024)	(0.025)	(0.205)	(0.177)
Private hospital		0.915***		0.844***		2.368***
		(0.086)		(0.082)		(0.512)
Public hospital		0.740***		0.735***		2.269***
		(0.114)		(0.108)		(0.272)
Public hospital x Policy		0.010		-0.062		-0.691**
		(0.050)		(0.056)		(0.288)
Private hospital x Policy		0.048		-0.061		-1.028***
		(0.049)		(0.050)		(0.350)
Age	-0.009***	-0.006***	-0.009***	-0.006***	-0.009***	-0.009***
	(0.002)	(0.002)	(0.002)	(0.002)	(0.002)	(0.003)
Age^2^	0.000***	0.000***	0.000***	0.000***	0.000***	0.000***
	(0.000)	(0.000)	(0.000)	(0.000)	(0.000)	(0.000)
Woman	-0.035***	-0.029**	-0.032***	-0.026**	-0.071***	-0.079***
	(0.011)	(0.011)	(0.011)	(0.011)	(0.025)	(0.027)
Ramadan	-0.022	-0.045	-0.028*	-0.046	0.010	-0.061
	(0.018)	(0.028)	(0.015)	(0.028)	(0.066)	(0.070)
Distance	0.009	0.021***	0.010	0.020***	0.061*	0.036**
	(0.013)	(0.003)	(0.011)	(0.003)	(0.031)	(0.015)
Visit	0.024***	0.021***	0.023***	0.020***	0.048***	0.039***
	(0.006)	(0.005)	(0.005)	(0.005)	(0.010)	(0.008)
Surgery	0.461***	0.474***	0.452***	0.464***	0.701***	0.707***
	(0.080)	(0.077)	(0.080)	(0.079)	(0.106)	(0.127)
Region FE	Yes	Yes	Yes	Yes	Yes	Yes
Month FE		Yes		Yes		Yes
Constant	12.320***	11.597***	4.976***	4.324***	2.647***	0.995*
	(0.187)	(0.093)	(0.166)	(0.084)	(0.207)	(0.523)
Observations	33,402	33,402	33,401	33,401	13,134	13,134
R-squared	0.128	0.395	0.133	0.353	0.233	0.317
Mean value (USD)^3^	327.22		290.58		34.05	

^1^*** p<0.01, ** p<0.05, * p<0.1. Robust standard errors in parentheses

^2^Note: Dependent variables in log transformations; Estimations include controls for type of hospital, gender, age, Ramadan and LoS. Standard errors clustered by hospital in parentheses. Policy is a dummy variable that indicates the period after the last policy change (from June 2016 onwards). Full table in Additional file 1: Section A1.3

^3^Mean values of the dependent variables in USD before logarithm transformation

That said, in relation to the original research questions, our findings show the introduction of a 10% cost-sharing component for secondary care is a potential instrument for redistributing demand, while it shows low effectiveness for containing costs for the provider. Moreover, other than the introduction of co-payments itself, there are several aspects of the policy implementation process that may be behind this result, from timings to lack of information. Nonetheless, to the extent that the data available allows us to show, the policy impact had very low (if any) impact in terms of costs for UNRWA.

Overall, the demand shift towards PRCS had a relevant impact for patients and healthcare services provision, but not for length of stay, even though UNRWA increased monitoring. These changes had contradictory impacts on costs and the overall effect on patient and UNRWA contribution was not significant.

## Conclusion

In this study, we examine the effect of introducing a 10% co-payment in hospitalization costs at private and public hospitals, using a natural experiment setting. We find that, after introducing the co-payment component, the probability of going to hospitals where coverage remained at 100% (PRCS) increased. Data demonstrate that the provider (UNRWA) costs did not change after the policy implementation and patients are choosing more the fully covered and cheaper hospitals.

Building on previous evidence, this study contributes to the contemporary debate on the net impact of implementing health care out-of-pocket payments in complex social and political contexts, such as the one of the Palestine refugees living in Lebanon. The analysis provides a general understanding on the demand for hospitalization in secondary care level and a thoughtful insight on how a particular policy change affected health care services from a *lessons learned* perspective. The outcomes of this project provide evidence on the characteristics and determinants of health care demand in UNRWA contracted hospitals, while indicating the magnitude and direction of the cost-sharing policy impact at different levels, enabling the identification of potential issues and advantages of this type of payment scheme in secondary care hospitalization.

According to the results, UNRWA introducing a cost-sharing component for private and public hospitals lead to a demand re-distribution towards PRCS hospitals, where treatment continued to be provided free of charge. This can mean an efficiency gain in case PRCS are able to answer to a higher demand, but also that access to private and public hospitals is now more restricted. We did not found a relevant impact on average LoS, but we found some evidence that the higher demand in PRCS happened more on longer stays, which is supported by our theoretical analysis. Moreover, because UNRWA increased control on the occupancy at the hospitals it is possible that both effects balanced out. If this is true, increasing control was effective and may have contributed to avoiding (or decreasing) system over-usage (overutilization of UNRWA services is frequently mentioned as a significant challenge for the health programme in official documents [32, 33]). For PRCS in particular, this provides some evidence that patients in more severe financial situations with more severe conditions were affected by the change in policy and face more constraints to chose public or private hospitals, even if it is their preferred option.

Comparing the evidence found with similar recent studies in different contexts, this type of policy in Low-Middle Income Countries (LMICs) and richer countries seems to have a similar pattern as in the Palestine refugee camps in Lebanon. In Kenya, a study showed that a voucher programme and the introduction of free maternity services (i.e., abolished payments - in the opposite direction of the policy implemented by UNRWA) were both positively associated with the continuous use of antenatal care. The authors conclude that policymakers should promote equity reducing financial barriers to care seeking patients [34]. Even in countries with more resources, paying for health care has a negative impact on utilization and effects are larger for the poorest [35, 36]. One potential difference may be that richer users or users from richer countries are able to compensate the initial decrease in healthcare consumption or spending, by consuming more later [37]. However, we believe this is not the case for PRL, since using data until more than one year after the policy was implemented, we still grasped the policy impact on the demand indicators. Relative to the RAND experience, which was an experimental study from 1974 to 1982 of health care use in the United States (the largest health policy study in U.S. history), a 10% increase in cost sharing would result in a 2% reduction in spending and the use of physician services was more responsive to cost sharing than use of hospital services. While in the PRL setting we are not studying a change in insurance scheme, users seem much more sensitive to changes in costs. It would be extremely interesting to study changes in user fees for primary care in UNRWA health centres and understand further if there are differences in the way refugees react to health care financing policy changes, to identify specific needs and behaviours of these communities [38, 39].

Although we found statistically significant correlations, with this data it is not possible to control for unobservable events happening during the analysis period that could affect the results - conflicts, natural disasters, political crises, etc. As such, one cannot assume direct causality of the policy impact. Additionally, another limitation of this study is the fact that there is not enough data on socio-demographics characteristics or patients benefiting from Social Safety Net (SSN) to understand exactly to what extent the cost-sharing policy is depriving poorest patients from quality health-care^5^. Finally, and probably most importantly, because we have a limited set of variables, to avoid endogeneity issues we had limited explanatory capacity, leaving a lot of unobservables in the error terms. We did not have access to characteristics of the disease, nor patients health outcomes nor measures of healthcare service quality. With this information we could have assessed better the policy impact, whether there were any signs of overcrowding (or crowding control measures) at PRCS hospitals before and after the policy being implemented. The lack of updated news and information on these hospitals also makes it more difficult to study these services.

Overall, the cost-sharing policy proved to be effective to re-distribute demand across hospital types, indicating that patients are generally price sensitive for secondary care hospitalization services. Nevertheless, the demand adjustment prevented UNRWA from containing more costs than before and the co-payment fee prevented extremely fragile patients from choosing their preferred hospital. This study provides UNRWA with an impact evaluation in-depth exercise, including valuable information on how their policies have an impact in terms of users behavior and cost containment strategies. We believe this analysis can be used for future reference in policy decision making and opens an important precedent of how research and institutions can work together to achieve a greater good for the target population.

## Supplementary Information

**Additional file 1** Supplementary proofs, maps, figures, tables.

## Data Availability

The data that support the findings of this study are available from The United Nations Relief and Works Agency for Palestine Refugees in the Near East (UNRWA) but restrictions apply to the availability of these data, which were used under license for the current study, and so are not publicly available. Data are however available from the authors upon reasonable request and with permission of UNRWA.

## Footnotes

1 Data were provided by UNRWA directly, but all opinions are the responsibility of the researcher.

2 The most common types of cost-sharing are: co-payments, payment of a fixed amount for each medical service; coinsurance, payment of a fixed percentage of the health care expenditure; and deductibles, payment of the first need of care each year [16].

3 Costs are fixed in the local currency, Lebanese Lira (LL), and then converted to USD at the exchange rate in place at the time – around 1500 LL for each USD. The exchange rate was quite stable during the period of analysis. During 2016 the rate varied from a max of 1,513.55 LL/USD to a minimum of 1,501.75 LL/USD. In 2017, there was even less variation.

4 With a total population we are not using the estimated average, but the true parameter. There is no need to estimate time-varying average treatment effects [25, 26].

5 SSN are patients supported by UNRWA who typically do not pay for healthcare, even after the policy changes. While we do not have information directly on who is under SSN programme, we excluded from the analysis all patients that always paid 0 for healthcare in our dataset
