# Supplementary material for: Co-payments and equity in care: enhancing hospitalisation policy for Palestine refugees in Lebanon
Source: BMC Health Serv Res. 2022 Jan 29;22:121. doi: 10.1186/s12913-021-07427-8 (PMC8800277; doi:10.1186/s12913-021-07427-8)
Supplement: Supplementary file 1 — Additional file 1 Supplementary proofs, maps, figures, tables. [file 12913_2021_7427_MOESM1_ESM.pdf]

A1 Additional Files

A1.1 Additional file 1 — Proofs

Proposition 1

Consider condition 1:

$$\begin{aligned} B(\eta) \geq S_2 C_2(\eta) \Leftrightarrow \\ \Leftrightarrow B(\eta) - S_2 C_2(\eta) \geq 0 \Leftrightarrow \\ \Leftrightarrow f(S_2, q) \geq 0 \Rightarrow f(S_2, \eta^*) = 0 \end{aligned} \tag{1}$$

Then, by the application of the Implicit Function Theorem: If f : $R^m \times R \Rightarrow R$ is a C^1 function, $f(x_0; y_0) = 0$, and $\frac{\partial f}{\partial x} \neq 0$, then for some neighborhood $U \subset R^m$ of (x_0) there is a C^1 function $g : U \Rightarrow R$ such that $g(x_0) = y_0$ and $f(x, g(x)) = 0$ for all $x \in U$. The partial derivatives of g at x_0 are given by the formula:

$$\frac{\partial g}{\partial x^i}(x) = -\frac{\frac{\partial f}{\partial x^i}(x_0, y_0)}{\frac{\partial f}{\partial y}(x_0, y_0)}$$

Then,

$$\begin{aligned} \frac{\partial \eta^*}{\partial S_2}(S_2) = -\frac{\partial f(S_2, \eta^*)/\partial S_2}{\partial f(S_2, \eta^*)/\partial \eta^*} \Leftrightarrow \\ \Leftrightarrow \frac{\partial \eta^*}{\partial S_2}(S_2) = \frac{C_2(\eta^*)}{\partial B(\eta^*)/\partial \eta^* - S_2(\partial C_2(\eta^*)/\partial \eta^*)} > 0 \end{aligned} \tag{2}$$

According to this relationship, the threshold for patients to get treatment is positively related to the price of the low quality hospital. This is, as the patients' contribution share increases, more severity is needed for patients to get treatment.

Proposition 2

Consider condition 2:

$$\begin{aligned} \theta B(\eta) - S_1 C_1(\eta) \geq B(\eta) - S_2 C_2(\eta) \Leftrightarrow \\ \Leftrightarrow (\theta - 1) B(\eta) - S_1 C_1(\eta) + S_2 C_2(\eta) \geq 0 \Leftrightarrow \\ \Leftrightarrow g(S_1, S_2, \eta) \geq 0 \Rightarrow g(S_1, S_2, \eta^{**}) = 0 \end{aligned} \tag{3}$$

Once again, by the application of the Implicit Function Theorem:

$$\begin{aligned} \frac{\partial \eta^{**}}{\partial S_1}(S_1, S_2) = -\frac{\partial g(S_1, S_2, \eta^{**})/\partial S_1}{\partial g(S_1, S_2, \eta^{**})/\partial \eta^{**}} \Leftrightarrow \\ \Leftrightarrow \frac{\partial \eta^{**}}{\partial S_1}(S_1, S_2) = \frac{C_1(\eta^*)}{\partial B(\eta^{**})/\partial \eta^{**} - S_1(\partial C_1(\eta^{**})/\partial \eta^{**})} > 0 \end{aligned} \tag{4}$$

Proposition 3

Consider condition 2:

$$\begin{aligned} \frac{\partial \eta^{**}}{\partial S_2}(S_1, S_2) = -\frac{\partial g(S_1, S_2, \eta^{**})/\partial S_2}{\partial g(S_1, S_2, \eta^{**}))/\partial \eta^{**}} \Leftrightarrow \\ \Leftrightarrow \frac{\partial \eta^{**}}{\partial S_2}(S_1, S_2) = -\frac{C_2(\eta^{**})}{\partial B(\eta^{**})/\partial \eta^{**} + S_2(\partial C_2(\eta^{**})/\partial \eta^{**})} < 0 \end{aligned} \tag{5}$$

Proposition 4

Consider the number of patients going to hospital 1 and 2 as:

$$W_1 = \int_{\eta^{**}}^{\bar{\eta}} f(\eta) d\eta \tag{6}$$

$$W_2 = \int_{\eta^*}^{\eta^{**}} f(\eta) d\eta \tag{7}$$

Then, taking into account the above relationships:

$$\begin{aligned} \frac{\partial W_1}{\partial S_1} &= -\frac{\partial \eta^{**}}{\partial S_1} f(\eta^{**}) < 0; \\ \frac{\partial W_1}{\partial S_2} &= -\frac{\partial \eta^{**}}{\partial S_2} f(\eta^{**}) > 0 \end{aligned} \tag{8}$$

And:

$$\begin{aligned} \frac{\partial W_2}{\partial S_1} = \frac{\partial \eta^{**}}{\partial S_1} f(\eta^{**}) - \frac{\partial \eta^{*}}{\partial S_1} f(\eta^{*}) = \frac{\partial \eta^{**}}{\partial S_1} f(\eta^{**}) > 0; \\ \frac{\partial W_2}{\partial S_2} = \frac{\partial \eta^{**}}{\partial S_2} f(\eta^{**}) - \frac{\partial \eta^{*}}{\partial S_2} f(\eta^{*}) < 0 \end{aligned} \tag{9}$$

If average costs are given by:

$$Av.Costs_1 = \frac{\int_{\eta^{**}}^{\bar{\eta}} C(\eta) f(\eta) d\eta}{\int_{\eta^{**}}^{\bar{\eta}} f(\eta) d\eta},\ C'(\eta) > 0 \tag{10}$$

$$Av.Costs_2 = \frac{\int_{\eta^{*}}^{\eta^{**}} C(\eta) f(\eta) d\eta}{\int_{\eta^{*}}^{\eta^{**}} f(\eta) d\eta},\ C'(\eta) > 0 \tag{11}$$

Applying the same reasoning:

$$\frac{\partial Av.Costs_1}{\partial S_1} > 0; \frac{\partial Av.Costs_1}{\partial S_2} < 0; \quad \frac{\partial Av.Costs_2}{\partial S_1} < 0; \frac{\partial Av.Costs_2}{\partial S_2} > 0 \tag{12}$$

A1.2 Additional file 2 — Maps

figure_s1.png

Figure S1 Hospitals by enumeration area

A1.3 Additional file 3 — Tables

A1.3.1 Robustness checks - Part I

Trying to further understand the results, in order to also better ensure their reliability, we performed a series of robustness checks to the estimations. These exercises consist in using different clustering methods, different policy shifts, dependent variables and model specifications.

Our main set of robustness checks presents the main estimations, but ignoring potentially endogeneity issues.

Table S1 shows our main estimation for hospital type demand, controlling for length of stay, bill value and UNRWA contribution. Results show an increase in demand for PRCS of 18 pp ($p < 0.01$), larger than the main result found. The variable for length of stay has opposite signs between PRCS and the other hospital types. Generally, average length of stay is higher for private hospitals, as more severe episodes require more resources. In fact, a simple mean test shows the average value is 0.7 days lower at PRCS and the difference is statistically significant at 1%. This said, the results from the multinomial estimation show a higher average stay in days at PRCS, as an increase in one day at the hospital makes it 2 pp more likely to choose a PRCS hospital. We believe this contradicting result comes from controlling for the variable bill value, which is highly correlated with length of stay (0.8) and is also expected to be correlated with disease severity. In this sense, when controlling for this factor, we can consider that length of stay becomes an indicator of efficiency. Since private hospitals are profit oriented, the length of stay should be closer to the optimal number of days necessary for each procedure, and thus lower compared to other type of facilities. As for Bill value and UNRWA contribution, the variables have the expected signs and are statistically significant, in PRCS hospitals interventions are cheaper and UNRWA contributes at 100% for all secondary care costs.

To control for non-linearities in cross-products, the interaction coefficients were computed following Karaca-Mandic et al. (2012). The interaction effect allows both the intercept and the marginal effect (slope) of UNRWA contribution and LoS on the expected probability of the dependent variable to be different before and after the policy was implemented. Due to the model non-linearity the marginal effect is not constant over its entire range. As such, the difference between the marginal effect in both moments gives the change in the conditional probability that the outcome variable is equal to one for a unit change in UNRWA contribution, as the co-payment share changes from zero to 10% (policy variable changes from 0 to 1). Regarding UNRWA contribution, the difference the marginal effect before and after the policy is implemented is not statistically significant, which was expected given that all patients were subject to the policy change. In turn, the difference in the marginal effect of LoS before and after the policy is negative for PRCS and positive for private. This means that the effect of staying 1 additional day at the hospital in the probability of going to a PRCS hospital was 0.7 pp lower after the policy was in place. At the same time, for private hospitals, one additional day hospitalized has a more positive impact on the probability of a patient choosing this hospital type, after the policy was implemented. As such, while demand increased at PRCS hospitals and decreased for private, staying longer became less likely to happen at PRCS facilities after June 2016. This may indicate that the increase in monitoring of LoS was successful in reducing inefficiencies at PRCS or that, due to the higher demand, services were forced to reduce hospitalization time for patients.

Table S2 is the equivalent to Table 4 in the main document, but with costs and LoS variables. Again we follow the previous evidence of a larger policy impact when we control for more variables. Introducing these new variables introduces a control for disease characteristics that can allows us to isolate further the policy impact. However, at the same time because demand, costs and length of stay are extremely correlated we cannot defend this result, as it can be biased by endogeneity issues.

In terms of visits frequency, as shown in S3, there is almost no difference in the policy impact compared to the main results (Table S3). Here we are working with much less observations and collapsed data, which can make a difference in the results variability.

Table S4 again shows that including the cost variables changes the results and we have a negative IRR value for stay in days in general after the policy was implemented. The interaction variables show shorter length of stays at public and private hospitals after the policy, which goes in line with the main result showing a decrease in shorter stays at PRCS - i.e. shorter stays shifted to public and private hospitals.

Regarding costs, even when including overlaping variables, we could not find a statistically signifficant impact on costs (Table S5).

Table S1 Policy impact estimation on demand for hospital type (Multinomial Logit - margins), from April 2016 to October 2017

	PRCS		Priv. Hospital		Pub. Hospital	
	(1.a)	(1.b)	(2.a)	(2.b)	(3.a)	(3.b)
Var. of interest:						
**Policy**	**0.035****	**0.180*****	**-0.018**	**-0.147*****	**-0.016**	**-0.033**
	**(0.015)**	**(0.061)**	**(0.017)**	**(0.032)**	**(0.013)**	**(0.065)**
Stay in days	-0.055***	0.022***	0.035***	-0.017***	0.019	-0.005
	(0.016)	(0.008)	(0.013)	(0.006)	(0.015)	(0.005)
Surgery	-0.005	0.098**	0.017	-0.048	-0.012	-0.050
	(0.041)	(0.043)	(0.040)	(0.030)	(0.026)	(0.031)
UNRWA contribution		2.880***		-1.819***		-1.061*
		(0.678)		(0.404)		(0.633)
Bill value		-3.001***		1.880***		1.121*
		(0.717)		(0.430)		(0.661)
UNRWA contr. (at p3==0)		5.745***		-3.547**		-2.198
		(2.226)		(1.582)		(1.621)
UNRWA contr. (at p3==0)		2.016*		-1.142		-0.874
		(1.097)		(0.725)		(0.602)
Difference		-3.26		2.405		1.324
Stay in days (at p3==0)		0.077***		-0.052***		-0.024
		(0.020)		(0.019)		(0.022)
Stay in days (at p3==1)		0.008		-0.007		-0.002
		(0.008)		(0.005)		(0.004)
Difference		0.069***		0.045**		0.022
Controls:						
Age	0.001	0.000	0.001	0.001**	-0.002	-0.001***
	(0.001)	(0.000)	(0.001)	(0.000)	(0.001)	(0.000)
Age^2	-0.000	0.000	-0.000	-0.000*	0.000	0.000
	(0.000)	(0.000)	(0.000)	(0.000)	(0.000)	(0.000)
Woman	0.003	-0.004	-0.002	0.002	-0.001	0.003
	(0.006)	(0.004)	(0.006)	(0.004)	(0.004)	(0.004)
Ramadan	-0.016	-0.002	0.012	0.004	0.004	-0.002
	(0.012)	(0.003)	(0.011)	(0.005)	(0.004)	(0.005)
Distance	0.035	0.034	0.023	0.097***	-0.058	-0.131**
	(0.058)	(0.033)	(0.040)	(0.029)	(0.096)	(0.056)
CLA	0.395	0.123**	-0.438	-0.153	0.043	0.030
	(0.317)	(0.055)	(0.301)	(0.109)	(0.116)	(0.109)
Visit	0.005	0.001	-0.005	-0.003	-0.001	0.001
	(0.005)	(0.001)	(0.006)	(0.002)	(0.002)	(0.001)
Observations	32,851	32,810	32,851	32,810	32,851	32,810

[1] Clustered standard errors by hospital in parentheses;*** p<0.01, ** p<0.05, * p<0.1;
[2] Dependent variables are a log transformation of the bill value, unrwa and patient contribution. Specification a does not contain costs related variables and avoids any potential issues of multicollinearity. Policy is a dummy variable that indicates the period of the policy change (from June 2016 onwards).

Table S2 Policy impact estimation on demand for PRCS hospitals (Multinomial logit - margins), different periods

	(1)	(2)	(3)	(4)
Policy - Jan		0.304***	0.263***	0.267***
		(0.040)	(0.052)	(0.008)
**Policy - Jun**	**0.227*** **	**0.166*** **	**0.158*** **	**0.157*** **
	**(0.026)**	**(0.041)**	**(0.040)**	**(0.004)**
UNRWA Contr.	4.686***	3.249***	3.060***	3.091***
	(0.494)	(0.464)	(0.555)	(0.041)
Stay in days	0.071***	0.050***	0.025***	0.024***
	(0.009)	(0.012)	(0.009)	(0.002)
Bill Value	-5.061***	-3.531***	-3.214***	-3.242***
	(0.472)	(0.486)	(0.593)	(0.044)
Age	0.001**	0.001*	0.000	0.000***
	(0.000)	(0.001)	(0.000)	(0.000)
Age^2	0.000	0.000	0.000	0.000*
	(0.000)	(0.000)	(0.000)	(0.000)
Woman	-0.003	0.001	-0.003	-0.003
	(0.007)	(0.003)	(0.003)	(0.002)
Ramadan	0.026***	0.006	-0.004	-0.001
	(0.010)	(0.006)	(0.004)	(0.005)
Distance	0.045***	0.042	0.038	0.037***
	(0.003)	(0.046)	(0.039)	(0.001)
CLA	0.480***	0.253**	0.146**	0.146***
	(0.031)	(0.104)	(0.068)	(0.006)
Visit	0.011**	0.006*	0.001	0.002***
	(0.004)	(0.003)	(0.001)	(0.001)
Surgery	0.284***	0.200***	0.117***	0.116***
	(0.024)	(0.035)	(0.041)	(0.007)

[1] *** p<0.01, ** p<0.05, * p<0.1. Robust standard errors in parentheses.

[2] (1) 2 months pre and post policy
(2) 5 months pre and post policy
(3) Full sample - January 2016 to October 2017
(4) Full sample with month FE - January 2016 to October 2017

[3] Note: The dependent variables are binary variables with the value 1 if the patient is at each hospital type and 0 otherwise. Note that all patients get treatment, thus for each observation at least one option must be selected. Coefficients show average marginal effects for multinomial logit regression results. Policy is a dummy variable that indicates the period after the last policy change (from June 2016 onwards). These model specifications control for individual and hospital specific variables.

Table S3 Policy impact on the demand volume of visits, by week

	Freq by 1000 PRL
PRCS	1.089***
	(0.162)
Policy - Jan	-0.521***
	(0.120)
**Policy Jan x PRCS**	**0.550*** **
	**(0.178)**
Policy - Jun	-0.215*
	(0.115)
**Policy Jun x PRCS**	**0.268* **
	**(0.148)**
Age	-0.004**
	(0.002)
Distance	0.264***
	(0.025)
Ramadan	-0.043
	(0.109)
CLA	-1.466***
	(0.049)
UNRWA Contr.	-0.256
	(0.200)
Stay in days	-0.045***
	(0.015)
Bill Value	0.757***
	(0.200)
Month FE	Yes
Year FE	Yes
Constant	-8.080***
	(1.588)
Observations	632
R-squared	0.756

[1] *** p<0.01, ** p<0.05, * p<0.1. Robust standard errors in parentheses.
[2] Full sample with month FE (January 2016 to October 2017);
[3] Note: We collapsed data to week level and normalized the hospital volume by the estimated number of PRLs in 2016 (280,000). The outcome variable is the number of hospital visits per 1,000 PRLs. Policy Jan is a dummy variable that indicates the period after the first policy change (from January to March 2016 onwards); and Policy Jun indicates the last version implemented in June 2016.

Table S4 Policy 3 impact estimation on Stay in Days (Neg. Binomial/Mult. Poisson - IRR), from April 2016 to October 2017 (with controls)

	Nbreg		Mult. Poisson
	(1)	(2)	(3)
Var. of interest:			
**Policy 3**	**0.996**	**0.860*** **	**0.906*** **
	**(0.029)**	**(0.021)**	**(0.020)**
Surgery	0.716***	0.678***	0.694***
	(0.061)	(0.057)	(0.060)
UNRWA contr. × Policy 3		1.001***	1.000***
		(0.000)	(0.000)
Priv. Hosp × Policy 3		0.778***	0.851***
		(0.051)	(0.049)
Pub. Hosp. × Policy 3		0.763*	0.810
		(0.123)	(0.127)
Private hospital		1.363***	1.389***
		(0.105)	(0.113)
Public hospital		1.451***	1.444**
		(0.205)	(0.210)
Controls:			
Age	0.986***	0.994***	0.993***
	(0.003)	(0.001)	(0.002)
Age squared	1.000***	1.000***	1.000***
	(0.000)	(0.000)	(0.000)
Woman	0.968**	0.971**	0.964**
	(0.013)	(0.012)	(0.018)
Ramadan	0.976	0.971*	0.960
	(0.021)	(0.016)	(0.032)
Visit	1.033***	1.017***	1.023***
	(0.011)	(0.006)	(0.006)
Distance	1.008	1.007***	1.011***
	(0.008)	(0.002)	(0.002)
Area		1.014	1.056
- CLA			
		(0.053)	(0.076)
- NLA		1.195***	1.308***
		(0.049)	(0.060)
-Saida		1.110**	1.227***
		(0.052)	(0.066)
- Tyre		0.896***	0.944*
		(0.024)	(0.032)
Constant	2.526***	2.194***	1.758***
	(0.039)	(0.054)	(0.097)
Observations	32,851	32,811	33,402

[1] *** p<0.01, ** p<0.05, * p<0.1. Robust standard errors in parentheses.

[2] Note: Coefficients show Incidence Rate Ratios (IRR) for a negative binomial and multinomial poisson regression results. Standard errors clustered by hospital in parentheses. Policy 3 is a dummy variable that indicates the period after the last policy change (from June 2016 onward).

Table S5 Policy 3 impact estimation on patient contr, UNRWA contr. and Bill value (OLS), from April 2016 to October 2017

	Bill value		UNRWA contr.		Patient contr.	
	(1.a)	(1.b)	(2.a)	(2.b)	(3.a)	(3.b)
Var. of interest:						
**Policy 3**	**-0.007**	**-0.001**	**-0.040****	**0.002**	**-0.134**	**0.091**
	**(0.013)**	**(0.007)**	**(0.019)**	**(0.006)**	**(0.102)**	**(0.116)**
Stay in days	0.199***	0.011**	0.193***	0.001	0.191***	-0.031**
	(0.021)	(0.004)	(0.021)	(0.002)	(0.019)	(0.014)
Surgery	0.605***	0.024**	0.590***	0.010	0.935***	-0.082
	(0.085)	(0.010)	(0.086)	(0.010)	(0.095)	(0.060)
Private hosp.	0.732***	0.076*	0.602***	-0.036	1.511**	0.569
	(0.049)	(0.039)	(0.041)	(0.033)	(0.558)	(0.390)
Public hosp.	0.601***	0.007	0.504***	0.024	1.110**	0.615*
	(0.058)	(0.013)	(0.055)	(0.018)	(0.525)	(0.302)
Bill value				0.960***		1.220***
				(0.013)		(0.058)
UNRWA contr.		0.983***				
		(0.011)				
Stay in days ×		-0.002		0.001		0.015
		(0.003)		(0.003)		(0.015)
Priv. Hosp × policy 3		0.148***		-0.109***		0.273
		(0.014)		(0.016)		(0.405)
Pub. Hosp × policy 3		0.118***		-0.085***		0.113
		(0.010)		(0.014)		(0.381)
Controls:						
Age	-0.001	0.000**	-0.001	-0.000**	0.000	0.003**
	(0.001)	(0.000)	(0.001)	(0.000)	(0.002)	(0.001)
Age squared	0.000***	-0.000*	0.000***	0.000**	0.000***	-0.000**
	(0.000)	(0.000)	(0.000)	(0.000)	(0.000)	(0.000)
Woman	-0.019**	-0.002	-0.017	0.001	-0.049**	-0.011
	(0.009)	(0.002)	(0.010)	(0.002)	(0.019)	(0.010)
Ramadan	-0.021*	0.004	-0.026**	-0.006	0.007	0.041
	(0.011)	(0.005)	(0.010)	(0.005)	(0.056)	(0.038)
Distance	0.012***	0.001**	0.011***	-0.000	0.010	0.000
	(0.002)	(0.000)	(0.002)	(0.000)	(0.012)	(0.005)
Visit	0.006*	0.001	0.005*	-0.000	0.019**	0.008*
	(0.003)	(0.001)	(0.003)	(0.001)	(0.009)	(0.005)
Area	0.389***	0.066***	0.328***	-0.045***	0.581	-0.162
- CLA						
	(0.099)	(0.015)	(0.092)	(0.013)	(0.422)	(0.284)
- NLA	0.455***	0.043***	0.418***	-0.018	0.672***	0.045
	(0.064)	(0.014)	(0.060)	(0.014)	(0.125)	(0.069)
-Saida	0.387***	0.047**	0.345***	-0.026*	0.740***	0.157**
	(0.069)	(0.018)	(0.058)	(0.014)	(0.163)	(0.072)
- Tyre	0.268***	0.031**	0.241***	-0.016	0.296***	-0.021
	(0.043)	(0.011)	(0.039)	(0.010)	(0.053)	(0.027)
Constant	11.325***	7.328***	4.100***	-6.810***	0.975*	-12.743***
	(0.065)	(0.047)	(0.055)	(0.162)	(0.550)	(0.656)
Observations	32,811	32,810	32,810	32,810	12,875	12.875
R-squared	0.702	0.983	0.675	0.982	0.596	0.900

[1] *** p<0.01, ** p<0.05, * p<0.1. Standard errors clustered by hospital in parentheses.

[2] Note: Dependent variable is in log transformation; Policy 3 is a dummy variable that indicates the period after the last policy change (from June 2016 onward).

A1.3.2 Robustness checks - Part II
In this section instead of replicating the main estimations with different specifications we perform further checks, with different models and data.
First we estimate the probability of changing hospital and assess how those patients that shifted to a PRCS after the policy change are affecting the results. We created a binary variable for when patients go to a private or public hospital in the first visit before June 2016, but change to a PRCS hospital for the second or further visit after that same date. Note that only 297 patients changed to a PRCS hospital, which corresponds to 1.10% of the sample and does not give us enough power to achieve rigorous estimates. Nevertheless, it can be useful to learn more about this small sample.
Results in Table S6 show that staying one more day hospitalised, slightly (but significantly) decreases the probability of changing to a PRCS hospital. This indicates that people that changed hospital had longer stays on average, which can relate to the theory that patients with more severe conditions - that take longer to treat - have more difficulties covering for the increase in costs. After the policy, patients are 3% more likely to change to a PRCS hospital, highlighting the previous result that demand for PRCS increased with the introduction of the cost-sharing component.

Table S6 Policy impact estimation on probability of changing hospital type (Probit - margins), from April 2016 to October 2017

	(1)<br>Change
Var. of interest:	
**Policy**	**0.029*****
	**(0.010)**
Stay in days	-0.004***
	(0.001)
Controls:	
Age	-0.001***
	(0.000)
Age^2	0.000***
	(0.000)
Woman	-0.006
	(0.006)
Visit	-0.005
	(0.003)
Surgery	0.011
	(0.008)
Area	
-CLA	-0.003
	(0.008)
-NLA	0.052**
	(0.020)
-Saida	0.040***
	(0.009)
-Tyre	0.049**
	(0.021)
Observations	5,823

[1] *** p<0.01, ** p<0.05, * p<0.1. Robust standard errors in parentheses.
[2] Note: Dependent variable is a binary variable, equal to 1 if the patient changed from a public or private hospital to a PRCS after the second visit. Policy 3 is a dummy variable that indicates the period after the last policy change (from June 2016 onward)..

The following estimations replicate the main exercise using data between January and May 2016. This aims at capturing the shift between the first policy change (January to March 2016) and the negotiations period (April to May 2016). During this *first policy*, patients had to cover 5% of their costs for secondary care in PRCS hospitals, 10% in public and 15% in private, whereas during the negotiations, secondary care was free of charge at all hospitals (as it was for the main estimations). We consider the first three months as the policy 1 and estimate its impact using exactly the same model and estimation strategies as before.

Table S7 to S9 show the results for the three main impact estimations: LoS, hospital demand by hospital type and costs. The results obtained in this section confirm our previous reasoning, in the sense that patients are price sensitive and when they have to cover for a larger share of the costs, demand increases at the facilities where that share is smaller, which also follows the previously developed theoretical framework. Length of stay was higher in public hospitals during policy 1 but significantly lower in private hospitals. Going to a private hospital when policy 1 was in place decreased the rate of stay in days by 0.24, meaning that patients were going less and for shorter stays, as in the case of policy 3 (table S7). Demand for PRCS hospitals was 20% during policy 1, which meant a decrease of almost the same magnitude in the demand for private hospitals, as showed in table S8. In terms of costs, again there is no direct impact of the policy at any level. UNRWA contribution decreased in policy 1 for private and public hospitals, and length of stay during policy 1 affected negatively the bill value, which is mot likely related to the average LoS decrease in the most expensive hospitals. The results for policy 1 follow the general evidence found for policy 3, which was in place after June 2016 and charges 10% of secondary care costs at private and public hospitals.

Table S7 Policy 1 impact estimation on Stay in days (LoS), from January to May 2016

	(1)<br>Stay in days
Var. of interest:	
**Policy 1**	**1.001*****<br>**(5.19e-05)**
Policy 1 × UNRWA cont.	0.821**<br>(0.065)
Policy 1 × Priv. Hosp.	0.740**<br>(0.11)
Policy 1 × Pub. Hosp.	1.341***<br>(0.11)
Private hospital	1.478***<br>(0.20)
Public hospital	0.968<br>(0.084)
Controls:	
Age	0.993***<br>(0.002)
Age^2	1.000***<br>(1.63e-05)
Woman	0.991<br>(0.020)
Ramadan	1.059***<br>(0.012)
Visit	0.682***<br>(0.060)
Surgery	0.909***<br>(0.016)
Area	
- CLA	1.126<br>(0.096)
- NLA	1.015<br>(0.088)
- Saida	0.872*<br>(0.068)
lnalpha	1.982***<br>(0.17)
Observations	8,295

[1] *** p<0.01, ** p<0.05, * p<0.1. Robust standard errors in parentheses.
[2] Note: Dependent variable is a count variable equivalent to the number of days each patient stayed at the hospital in each visit. Policy 1 is a dummy variable that indicates the period of the 1st policy change (from January to March 2016).

Table S8 Policy 1 impact estimation on hospitals demand, from January to May 2016

	(1) PRCS	(2) Private	(3) Public
Var. of interest:			
**Policy 1**	**0.202****	**-0.170****	**-0.032**
	**(0.095)**	**(0.077)**	**(0.030)**
Policy 1 × UNRWA contribution	-0.002***	0.002***	0.001
	(0.001)	(0.001)	(0.001)
Policy 1 × Stay in days	0.208***	-0.144***	-0.064
	(0.043)	(0.037)	(0.049)
Stay in days	-0.048***	0.027**	0.021
	(0.011)	(0.012)	(0.015)
Controls:			
Age	0.002	0.001	-0.003
	(0.002)	(0.001)	(0.002)
Age^2	-0.000	-0.000	0.000
	(0.000)	(0.000)	(0.000)
Woman	0.016*	-0.011	-0.005
	(0.008)	(0.009)	(0.003)
Distance	0.042	0.030	-0.072
	(0.064)	(0.042)	(0.105)
Surgical	0.181***	-0.112***	-0.069
	(0.043)	(0.039)	(0.060)
Observations	8,295	8,295	8,295

[1] *** p<0.01, ** p<0.05, * p<0.1. Robust standard errors in parentheses.

[2] Note: The dependent variables are binary variables with the value 1 if the patient is at each hospital type and 0 otherwise. Note that all patients get treatment, thus for each observation at least one option must be selected. Coefficients show average marginal effects for multinomial logit regression results. Standard errors clustered by hospital in parentheses. Policy 1 is a dummy variable that indicates the period of the 1st policy change (from January to March 2016).

Table S9 Policy 1 impact estimation on costs, from January to May 2016

	(1) Bill value	(2) UNRWA Contr.	(3) Patient Contr.
Var. of interest			
**Policy 1**	**0.052**	**-0.039**	**-0.214**
	**(0.049)**	**(0.044)**	**(0.253)**
Policy 1 × UNRWA cont.	0.000***		0.001***
	(0.000)		(0.000)
Policy 1 × Stay in days	-0.063**	0.002	-0.019
	(0.025)	(0.021)	(0.040)
Private hospital	0.680***	0.616***	1.840***
	(0.058)	(0.063)	(0.298)
Public hospital	0.538***	0.544***	1.918***
	(0.053)	(0.046)	(0.288)
Policy 1 × Priv. Hosp	-0.059	-0.092**	0.146
	(0.036)	(0.043)	(0.298)
Policy 1 × Pub. Hosp	-0.040	-0.086**	-0.424
	(0.057)	(0.039)	(0.281)
Stay in days	0.215***	0.209***	0.156***
	(0.029)	(0.029)	(0.030)
Controls:			
Age	0.001	0.000	0.001
	(0.001)	(0.001)	(0.001)
Age^2	0.000***	0.000***	0.000*
	(0.000)	(0.000)	(0.000)
Woman	-0.035***	-0.036***	-0.050**
	(0.011)	(0.010)	(0.022)
Area			
- CLA	0.135	0.112	-0.011
	(0.131)	(0.128)	(0.140)
- NLA	0.205*	0.200*	0.200
	(0.117)	(0.113)	(0.121)
- Saida	0.127	0.118	0.157
	(0.120)	(0.114)	(0.121)
- Tyre	0.094	0.096	0.001
	(0.114)	(0.113)	(0.116)
Surgical	0.505***	0.550***	0.409***
	(0.064)	(0.082)	(0.062)
Constant	11.582***	4.271***	1.633***
	(0.124)	(0.121)	(0.272)
Observations	8,295	8,295	4,628

[1] *** p<0.01, ** p<0.05, * p<0.1. Robust standard errors in parentheses.
[2] Note: Dependent variables are a log transformation of the bill value, unrwa and patient contribution. Policy 1 is a dummy variable that indicates the period of the 1st policy change (from January to March 2016).

The last robustness check, presented in table S10 uses the year of 2017 as proxy for a control year. Facing one of the greatest limitations of this project - not being able to evaluate the before and after trends in a control group of people from the same context - in this estimation we perform the same regression as the main model in Table 4 of the main document, but with data from 2017 and thus considering a fictional policy change in June 2017. We assume patients in 2017 are equivalent to the patients in 2016, thus a potential control group, that was not subject to a policy change. These results help confirming that the main estimation is not grasping an effect of seasonality associated with the month of June, despite all the control variables. All policy indicators are not significant. Length of stay at private hospitals after June 2017 seems to decrease with a statistically significant impact, but the coefficient is really close to zero (marginal effect of 0.6pp).

Table S10 (Fake) Policy impact estimation on hospital demand, from January to October 2017

	(1) PRCS	(2) Priv. Hosp.	(4) Pub Hosp
Var. of interest			
**Policy**	**-0.001**	**0.001**	**0.000**
	**(0.002)**	**(0.003)**	**(0.003)**
UNRWA contribution	1.753	-1.009	-0.744
	(1.126)	(0.702)	(0.569)
Bill value	-1.790	1.019	0.771
	(1.163)	(0.721)	(0.587)
Stay in days	0.005	-0.006*	0.001
	(0.006)	(0.004)	(0.003)
Surgery	0.043	-0.022	-0.021
	(0.044)	(0.025)	(0.026)
UNRWA contr. (at p4==0)-(at p4==1)	0.038	0.008	0.005
Stay in days (at p4==0)-(at p4==1)	-0.001	-0.001	0.001
Controls			
Age	0.000	0.000	-0.000
	(0.000)	(0.000)	(0.000)
Age^2	0.000	-0.000	-0.000
	(0.000)	(0.000)	(0.000)
Woman	-0.005	0.000	0.004
	(0.005)	(0.005)	(0.005)
Ramadan	-0.004	-0.001	0.005
	(0.003)	(0.004)	(0.004)
Distance	0.023	0.105***	-0.128***
	(0.025)	(0.022)	(0.035)
CLA	-0.002	-0.054	0.056
	(0.027)	(0.112)	(0.096)
Visit	0.000	-0.003	0.002
	(0.001)	(0.003)	(0.003)
Observations	17,524	17,524	17,524

[1] *** p<0.01, ** p<0.05, * p<0.1. Robust standard errors in parentheses.
[2] Note: The dependent variables are binary variables with the value 1 if the patient is at each hospital type and 0 otherwise. Note that all patients get treatment, thus for each observation at least one option must be selected. Coefficients show average marginal effects for multinomial logit regression results. Standard errors clustered by hospital in parentheses. Policy is a dummy variable that indicates the period of the 3rd and main policy change if it had happened in 2017 (from June 2017 onward).
